# Efficacy of Psychological Interventions on Depression Anxiety and Somatization in Migrants: A Meta-analysis

**DOI:** 10.1007/s10903-020-01055-w

**Published:** 2020-07-25

**Authors:** Daniela Sambucini, Paola Aceto, Edvaldo Begotaraj, Carlo Lai

**Affiliations:** 1 Department of Dynamic and Clinical Psychology, Sapienza University of Rome, Via degli Apuli, 1, 00185 Rome, Italy; 2 A. Gemelli University Polyclinic, IRCSS Foundation, Rome, Italy; 3 Sacred Heart Catholic University, Rome, Italy

**Keywords:** Migration, Psychological intervention, Depression, Anxiety, Somatization

## Abstract

Many studies reveal the effectiveness of different psychological interventions on the adult refugees reporting mental health distress. Aim of this metanalysis was to test the efficacy of different psychological treatments on the depressive, anxiety and somatization symptoms on refugees and asylum seekers. Fifty-two studies, since 1997 to 2019, were included in the systematic review and 27 of those were included in the metanalysis. Studies providing a pre and post treatment methodological design were included. All treatments reported significant effects on the three outcomes. Qualitative observations showed a probability to have a significant pre-post treatment effects on trials with outcome of depression (56%), anxiety (44%), and somatization (42%). Cognitive behavioral treatment resulted the most effective treatment. The status of refugee compared to the status of asylum seeker seems to have a great effect on the effectiveness of the treatment.

**Electronic supplementary material:**

The online version of this article (10.1007/s10903-020-01055-w) contains supplementary material, which is available to authorized users.

## Introduction

Geopolitical migration caused the emergency to individuate an efficacy intervention for treating the traumatic symptomatology of the migrants [1]. The migrants often originate from countries with governate conditions of war or great poorness that increase the psychological distress [2, 3].

According UNHCR data of the 2015 the number of applications for asylum increased in Europe more than doubling from 2013 [4, 5]; in this period 17 million of refugees and asylum seekers were outside their counties of origin [6].

From 2013 to 2015 Italy State responded positively to about 40% of asylum applications, a percentage that was lowered in 2016 [7].

The number of people in the world who suffer from political violence and war pursued by the war seems to grow by about 1% every year [8].

The nature of conflict changed in last 20 years considering that the current war victims are civilians rather than combatants [3] causing a phenomenon of forced migration that represents, in many cases, the only option to survive. The forced migration, due to politics or war reasons produces unmistakable psychological signs [5]. Migrants experienced multiple stressful events before, during and after their travel: imprisonment, rape, ethnic cleansing, physical violence and torture. They often witnessed violence against or the death of loved ones [2]; persecutions, bereavement in their origin countries and discrimination in their host countries that contribute, successively, them to develop a “re-traumatization” post migration [5].

The exposure to interpersonal violence associated with emotional, sexual and physical abuse, torture and exploitation, and other atrocities committed in war, cause psychological distress that have strong impact on survivors life. Many psychological sequelae as destructive behaviors, substances misuse, self-harm, unsafe sexual practices and involvement in abusive relationship seems to be due to these traumatic conditions [9].

Moreover, forced migrants could experience deaths or suicides of their loved people, parents, kin, sons etc. These bereavements could expose the survivor migrant to thoughts of death, to a probability of exacerbation of the psychopathological symptoms that could last many years, and also to suicidal attempts. For this reason, it seems very important to explore what happens after the death or the suicide of a significant other, so to provide a proper and ad hoc care [10]. According to this clinical issue, it is important the knowledge of the neurobiological factors that underlie the suicide risk. Recent findings [11] showed an association between a biological dysregulation with the suicide attempts. This dysregulation has been interpreted as a compensatory mechanism that involves the prolactin and the thyroid hormones, useful to corrects the reduced central serotonin activity. In the next future this neurobiological correlate could be useful to individuate a suicide risk in the clinical practice [11]. The displacement’s perception, the events that caused the migration and the hostility in the host country post migration (culture shock), seem to decrease life expectancy, and produce insecurity, isolation and poorness, causing the development of mental illness including anxiety, depression and somatization [5].

In the last 20 years many types of psychological treatments—combined or not with pharmacological treatment—have been applied in migrants showing to be efficient [8–12].

Nowadays there are different systematic reviews that summarize the great number of studies on the efficacy of psychological intervention to post-migration symptoms. However, the four metanalysis published in the last ten years are focused on specific post-traumatic stress disorder PTSD outcome. The large number of studies published on this field allowed to perform metanalysis on specific therapeutic approaches and on specific psychological dimensions.

Aim of the present study was to assess, in adult migrants, the outcome of the main psychological interventions on post migration specific symptomatology: depression, anxiety and somatization.

## Method

### Search Strategy and Data Sources

The present systematic review and metanalysis has been registered on the International Prospective Register of Systematic Reviews (PROSPERO).

The literature search was conducted on health data base that included: Pubmed, Scholar, Psych INFO, Published International Literature on Traumatic Stress (PILOT). Additional studies were identified by cross-referencing.

### Inclusion Criteria and Selection of Studies

The search strategy was based on the following main search components: “migrants”, “refugees”, “asylum seekers” (alone and combined). All randomized controlled trials (RCT), multiple perspective cohort trials (MCT) and single perspective cohort trials (SCT) written in English and assessing psychological and/or pharmacological intervention with pre and post evaluation of depression, anxiety, somatization were considered eligible for inclusion. Only experimental groups investigating psychological interventions were considered for comparisons. The control groups were eliminated as they included waiting lists or usual treatments. Included studies had to report at least one quantitative measure of depression and/or anxiety and/or somatization assessed before and after treatment.

### Data Extraction and Coding

A qualitative systematic review for all 52 studies was performed (Table 1).

**Table 1:** Description of the 27/52 studies considered in the metanalysis

References	RCT	Control	Follow up	Samples	Subjects (total and for experimental samples pre and post. In order of samples)	Status	Provenance	Outcome	Measures	Experimental samples CBT treatments-NET-Psychodinamic therapy-Combined Psychological treatment (Combined psy)-Combined Pharmachological and psychological treatment (Combined pha)	Findings for each treatment applied outcome
[13]	Acarturk (2015)	Yes	1 mm	CBT (EMDR) WCL( no treatment)	29 15 14	Refugees	Siria	TEI PTSD Depression	IES-R BDI II	CBT (EMDR) 7 sessions, 90 min, weekly	PTSD Depression: significantly lower. remission maintained
[14]	Acarturk (2016)	Yes	1 mm	CBT (EMDR Focus recent trauma prolonged at present time) WCL (no treatment)	98 49/37 49/33	Refugees	Siria	TEI PTSD Depression Neuropsychiatric symptomatology	HTQ IES-R BDI-II HSCL 25 MINI	CBT (EMDR) 4 sessions	PTSD Depression Impact event significantly symptoms reduced, after 1 month maintained
[15]	Adenauer (2011)	Yes	4 mm	NET (Writed by therapists and reading high voice to subject) WLC (no treatment or antidepressants)	34 16/11 18/8	Refugees Asylum seekers		PTSD Depression Dystimya Neurocorrelates	MEG-ssVEF CSI CWDTE CAPS HAM-D MINI	NET 12 sessions, 108 min, weekly or biweekly	PTSD Depression significant effectiveness significant Changing of the neural correlates of the processing. NET increased occipital and parietal activity After 4 mm. maintained
[16]	Buhmann (2016)	Yes	2 mm 4 mm 6 mm 8 mm	CBT (commitment therapy and mindfulness and visual exposition) Combined Psy (CBT and psychoeducation and antidepressants Combined Pha (psychoeducation and sertraline or mianserine WLC (no treatment)	280 70/52 71/62 71/55 68	Refugees	Iraq Iran Lebanon Yugoslavia Afghanistan	PTSD Anxiety Depression Somatization Stress Panic InabilityPsychofisical	HTQ HSCL25 SCL90 HAM-D HAM-A VAS SDS WHO-5	CBT 16 sessions, weekly Combined psy 4 sessions, weekly Combined pha 8 sessions, weekly	Depression: treatment with antidepressant in combination psychoeducation was associated with significant decrease and significantly decrease in patients receiving medicine Anxiety: significant effect of medication
[17]	Carlsson (2018)	Yes		Combined Pha (CBT SM for acquire coping skills, relax, divided attention, behavioral activation) and pharmacological (mianserin or sertraline) and psychoterapy Combined Pha (CBT CR psychoeducation, cognitive rebuilding of negative ideas.) and pharmacological (mianserin or sertraline) and psychotherapy TAU	140 62/53 64/52	Refugees	Afghanistan Yugoslavia Iran Iraq Lebanon	PTSD Panic, Anxiety Depression Somatization Wellbeing Disability, Functioning; Symptomatology	HTQ HSCL 25 HAM D HAM A SCL VAS WHO SDS GAF S GAF F	Combined Pha (SM and CR) 24 sessions, and 10 sessions with medical doctor, 16 sessions with psychotherapist. 60 min biweekly	PTSD Somatization Functioning Disability: CR significant effect Depression Anxiety Somatization: SM significant effects Anxiety: beta significant
[18]	Hensel-Dittmann (2011)		4 ww 6 mm 12 mm	NET (Required by therapysts and corrected by subject with reading) CBT (SIT: cognitive behaviour semistructured intervention)	28 14/10 13/10	Asylum seekers		PTSD Depression Neurosymptomatology	CAPS HAM-D MINI	NET 10 sessions, 90 min CBT (SIT) 10 sessions	PTSD Sygnificatively reduction with NET Maintained at 6 and 12 mm
[19]	Hijazi Alaa (2014)	Yes	2 mm 4 mm	Brief NET (Write the therapists) WLC (no treatment)	63 41/39 22	Refugees	Iraq	PTSD Depression Posttraumatic growth Psychological wellbeing Symptomatology of stress posttraumatic Somatization	HTQ PTGI WHO5 BDI-II PHQ-15	Brief NET 3 sessions, 60–90 min, weekly	PTSD Depression Somatization: Reduction at 2 and 4 mm Well being improved at 2 and 4 mm
[20]	Hinton (2004)		22 ww	Combined Pha (Immediate CBT and SSRI, benzodiazepine, gabapentin) Combined Pha (Delayed CBT and SSRI, benzodiazepine, gabapentin)	24 12 12	Refugees	Vietnam	PTSD Depression Anxiety Panic Psychophysical symptomatology	HTQ ASI HSCL 25 NPASS OPASS	Combined Pha immediate CBT 11 sessions Combined Pha delayed CBT started after 11 sessions for another 11 sessions	PTSD Depression Anxiety: Improvement since 1 assessment to 2 assessment for immediate and RET and treatments. Assessment since 1 to 3 significant improvement Psychophysical symptomatology, improve significantly
[21]	Hinton (2005)		28 ww	Combined Pha(immediate CBT: and SSRI, Clonazepam, social support) Combined Pha (delayed CBT: and SSRI, Conazepam, social support)	40 20 20	Refugees	Cambogia	PTSD Anxiety Somatization	ASI CAPS N-PASS O-PASS N-FSS O-FSS SCL90R	Combined Pha immediate CBT 12 sessions Combined Pha delayed CBT after 12 sessions for another 12 sessions	PTSD Anxiety Panic Orthostatic parameters Flashbacks: Significant group effect, time effect and interaction Follow up: Significant time effect and group effect and time interaction On all measures Immediate group has significant lower scores at second assessment. Delayed CBT improvement at 3 assessment
[22]	Neuner (2010)	Yes	6 mm	NET (more of 8000 words with supervision) CBT (Stabilization or psychoactive medication and TFT)	32 16 16		Turkey Balkans Africa	Depression PTSD Pain	PDS CIDI HSCL25 CAPS VCOV	NET and CBT (Stabilization and TFT) 9 sessions, 120 min, weekly and biweekly	PTSD: Significant main effect time and time for treatment interaction. NET significant difference within and between on PTSD, also to 6 mm Pain:: Significant time for treatment interaction
[23]	Paunovic (2001)		6 mm	Combined Pha (CBT: Exposition and tricyclics and SSRIs) or bensodiazepines or neuroleptics or muscle relaxants or neuroleptic) Combined Pha (CBT: exposition and cognitive therapy and control breathing and tricyclics and SSRIs or bensodiazepines or neuroleptics or muscle relaxants or neuroleptic)	40 20 20	Refugees		PTSD Depression Anxiety Event impact Quality life	CAPS IV HAM D HAM A PSS-SR IES R BDI STAI-S QOLI WAS BAI ADIS	Combined Pha CBT Exposition, 8 sessions, 20–60 min, weekly CBT, 16–20 sessions, 60–120 min, weekly	PTSD Depression Anxiety Event impact Quality life: CBT significant, better at post test. Both efficacy within on all measure and maintained at follow up
[24]	Sonne (2016)	Yes		Combined Pha (CBT and manualized psychotherapy and social counselling and mindfullness and Sertraline max 75 mg) Combined Pha (CBT and manualized psychotherapy and social counselling and mindfulness and Venlafaxine max 50 mg) Pharmacological treatment (same Sertraline)	207 108/88 98/68	Refugees	Middle East	CPTSD Depression Anxiety Somatization	HTQ HAM D HAM A HSCL 25 SCL 90 SDS GAF GAS VAS CSS WHO5	Combined Pha 24 sessions, 10 sessions medical doctor, 16 sessions psychologist	CPTSD Depression Anxiety: Good results for SSRI Functioning: significant difference within for Sertraline and significant differences between for both SAS significant differences both within GAF S significant difference between
[25]	Stenmark (2013)	Yes	1 mm 6 mm	NET (no written) CBT (Focalization and help for psychological problem)	81 51/38 30/22	Refugees Asylum seekers	Middle East	PTSD Depression Neurological state	CAPS HAM D MINI	NET: 10 sessions, 90 min CBT (Focalization): 10 sessions 86 min	PTSD Depression: Both refugees and asylum seekers reduced their mental problems. Both treatment symptomatologichal reduction but more pronounced for NET. Between group at 6 mm no significant effects. CAPS and HAM D significant main effects of time
[26]	Ter Heide (2011)	Yes	3 mm	CBT (Stabilization: here and now on trauma memory) CBT (EMDR)	20 10/5 10/5	Refugees Asylum Seekers	Afghanistan Algeria Bosnia Turchia Angola Lebanon	PTSD Depression Anxiety Quality life Neuropsychiatric state	HTQ HSCL 25 WHOQOL brief SCDI 1 MINI 10	CBT (Stabilization and EMDR) 11 sessions, weekly and biweekly	PTSD: significant difference between conditions, EMDR some improvement. Significant differences between treatments for HSCL-25 (anxiety and depression) and WHOQOL, EMDR improvements
[27]	Wang (2017)	Yes	3 mm 6 mm	Combined Psy immediate (CBT psychotherapy, group therapy and multivitaminic) Combined Psy delayed: control (CBT psychoterapy, grouptherapy delayed after 3 mm and multivitaminic immediate)	34 13 15	Asilum seekers	Kosovo	PTSD Depression Anxiety Panic	HTQ HSCL 25 SF.MPQ WB FACES PRS WHODAS	Combined Psy CBT 10 sessions, 90 min, group therapy 10 session, 90 min weekly	PTSD: Significant the effect of intervention at 6 mm Pain: at 3 mm
	Multiple perspective cohort trials										
[28]	Drozdek (2010)	Yes	6 mm 12 mm	Combined Psy 3*3 Combined Psy 3*2 Combined Psy 2*2 Dynamic: 1*1 Control (Pharmachological) 5 phases: 1 norms, values of group treatment, psychoeducation, alliance, assessment of problems, treatment goals and symptoms 2 presentations, damage core beliefs, fear of loss control, guilt, shame, grief, acknowledgement, resilience 3 telling the trauma story, exposure and cognitive restructuring 4 reconnecting the present with past and future, damage core beliefs, roles and identity, coping strategies, current worries and future outlook, resilience 5 psychoeducation, relapse prevention, treatment evaluation, farewell ritual. (employed dinamic and behavior tecniques)	88 34 19 11 6 18	Refugees Asylum seekers	Iraq Iran Afghanistan	PTSD Depression Anxiety Stress Psychofisic symptoms Psychotic symptoms	HTQ HSCL-20 SLC-90 Psychoticism scale	Specifically: Group therapy 58 sessions, 90 min, daily No verbal therapy 58 sessions, 75 min, daily Combined Psy 3*3 No verbal psychotherapy, 3 sessions (psychomotor body therapy, art therapy, music therapy) Group therapy, 2 sessions, 3 days week Combined Psy 3*2 same thing of 3*3 but 2 days week) Combined Psy 2*2 psychotherapy 2 sessions Group therapy 1 sessions, 2 days week Dynamic 1*1 Support, 48 sessions, weekly	PTSD Depression Anxiety Psychoticism Combined 3*3 and 3*2 Significant effect within PTSD ( 2*2) Differences between not statistically significant
[29]	Drozdek (2012)	Yes	12 mm	Combined Psy 3*3 Combined Psy 3*2 Combined Psy 2*2 WCL (for 6 mm no treatment) 5 phases: 1 norms, values of group treatment, psychoeducation, alliance, assessment of problems, treatment goals and symptoms 2 presentations, damage core beliefs, fear of loss control, guilt, shame, grief, acknowledgement, resilience 3 telling the trauma story, exposure and cognitive restructuring 4 reconnecting the present with past and future, damage core beliefs, roles and identity, coping strategies, current worries and future outlook, resilience 5 psychoeducation, relapse prevention, treatment evaluation, farewell ritual. (employed dynamic and behavior techniques)	71 27 22 7 16	Refugees Asylum seekers	Iran, Afghaniztan	PTSD Depression Anxiety Stress Psychophysic symptoms	HTQ HSCL20 SLC90	Specifically: Group therapy 58 sessions, 90 min, daily No verbal therapy 58 sessions, 75 min, daily Combined Psy 3*3 No verbal psychotherapy, 3 sessions (psychomotor body therapy, art therapy, music therapy) Group therapy, 2 sessions, 3 days week Combined Psy 3*2 same thing of 3*3 but 2 days week) Combined Psy 2*2 psychotherapy 2 sessions Group therapy 1 sessions, 2 days week	PTSD Depression Anxiety Stress Psychophysics symptoms The treatments were significant but 2*2 not significant effect on PTSD No significant difference between treatments and treatments and control, but 3*3 and 3*2 more efficacy of 2*2
[30]	Lakshmi Vijayakumar (2017)	Yes	6 mm 15 mm	Dynamic (CASP: emotional support contact with a voluntary community and support of the planning cards at moment of distress. Medical doctor) Control (to provide the telephone number that can use for supportive help)	485 288/139 187	Refugees	Tamil Nadu (India)	Depression Suicide ideation PTSD Alcohol abuse	Beck SSI (in WHO) SUPRE MISS CESD-R AUDIT PCL	Dynamic (CASP) 60 sessions, 4 mm medical doctor	PTSD and Depression, significant differences between on, and reduce of 2 unites the means of the scores (significant within)
[31]	Weinstein (2016)	Yes		Dynamic (Need satisfaction Basic psychological need Relieves the frustrations linked to the autonomy competences and relationships needs) Control: no treatment	41 24 17	Refugees	Siria	Need frustration PTSD ditress Depression	Psychological need scale PSS CES-D STAI 17 items self reported	Dynamic (Need satisfaction) 7 sessions, 24 min, daily	Depression and stress decreased with one week long intervention but not significantly and alleviated need frustration
	Single perspective cohort trials										
[32]	Brune (2014)			Combined Pha refugees (psychodynamic and CBT and antidepressant, anxiolytic, hypnotic)) Combined Pha no legal status (psychodynamic and CBT and antidepressant, anxiolytic, hypnotic))	190 121 69	Refugees Asylum seekers	Yugoslavia America Latina Turchia Africa Iraq Russia	Depression Psychosocial distress	HAM-D CGI	Combined Pha daily, pharmacological treatment, 22 months	Depression: significant results at end therapy for refugees. Psychosocial distress: no significant differences within and between
[33]	Carlsson (2010)		9 mm 23 mm	Combined Psy (psychodynamic, social counselling, physiotherapy, medical assistance, some serotonin)	69/62	Refugees	Iraq	Depression Anxiety Distress PTSD Quality life Event impact	HTQ, HAM-D HSCL25 WHOQOL brief		Quality life: significant differences at 9 mm Anxiety: significant differences between 9 and 23 mm Significant differences at 23 mm for all measures in 1/3 patients
[34]	D’Ardenne (2007)			CBT refugees interpreter trauma focalization, exposition) CBT refugees no interpreter (trauma focalization, exposition) CBT asylum seekers (trauma focalization, exposition)	112 36 31 45	Asylum seekers Refugees		PTSD Event impact Depression Quality life	IES BDI Manchester short assessment quality life	CBT (focalization, exposition) 9.1 sessions, 60 min, weekly	Depression: significant differences for all groups, more in asylum seekers Event impact: significant differences for all groups, more for asylum seekers Quality life: significant differences for refugees no interpreter and asylum seekers
[35]	Drozdek (2014)		12 mm 24 mm	Combined Psy 3*3 Combined Psy 3*2 Combined Psy 2*2 2 nonverbal 5 phases: 1 norms, values of group treatment, psychoeducation, alliance, assessment of problems, treatment goals and symptoms 2 presentations, damage core beliefs, fear of loss control, guilt, shame, grief, acknowledgement, resilience 3 telling the trauma story, exposure and cognitive restructuring 4 reconnecting the present with past and future, damage core beliefs, roles and identity, coping strategies, current worries and future outlook, resilience 5 psychoeducation, relapse prevention, treatment evaluation, farewell ritual. (employed dynamic and behavior techniques)	69/66 (all)	Refugees Asylum seekers	Iran Afghanistan	PTSD Depression Anxiety	HTQ HSCL20	Combined Psy 85 sessions: 1 phase 10 sessions 2 phase 20 sessions 3 phase 10 sessions 4 phase 30 sessions 5 phase 15 sessions Specifically: Group therapy 85 sessions, 90 min, daily No verbal therapy 85 sessions, 75 min, daily Combined Psy 3*3 No verbal psychotherapy, 3 sessions (psychomotor body therapy, art therapy, music therapy) Group therapy, 2 sessions, 3 days week Combined Psy 3*2 same thing of 3*3 but 2 days week) Combined Psy 2*2 psychotherapy 2 sessions Group therapy 1 sessions, 2 days week Dynamic 1*1 Support, 1 sessions, weekly	PTSD, Anxiety, Depression significant reduction for 3*3 treatment Anxiety significant differences in refugees
[36]	Halvorsen (2010)		1 mm 6 mm	NET (no verbal exposition all traumatic event, the biography is recorded and corrected)	16	Refugees Asylum seekers	Afghanistan Eritrea Kosovo Etiopia Iran Sudan Togo Iraq	PTSD Depression	CAPS HAM-D Sociodemographic questionnaire	NET 3sessions	PTSD: significant difference pre-post treatment, pretreatment-follow up and post treatment-follow up Depression: significant difference between pretreatment- follow up Avoidance: significant difference pre-post treatment, pretreatment-follow up Hyperarousal: significant difference between pretreatment-follow up
[37]	Raghavan (2013)		6 mm 18 mm	Combined Pha (medicine program of survivors of torture: individual and group therapy, counselling, free medicine)	178/172	Refugees Asylum seekers	Europa America Africa	General symptoms PTSD Depression Anxiety Somatization	HTQ BSI	Combined Pha 7.5 sessions, daily	Depression Anxiety Somatization PTSD significant differences at 6 mm
[38]	Sierra Van Wyk (2012)		6,9 mm	Combined Psy (psychoeducation, structured skills based therapy, expressive therapy, supportive therapy, couples and family therapy, CBT, exposition)	70/62	Refugees	Burma	PTSD Depression Anxiety Somatization	HTQ HSCL 37 Post Migration Living Difficulties Checklist	Combined Psy 2–3 sessions, 120–150 min	PTSD Depression Anxiety Somatization: Significant decrease symptoms
[39]	Whitsett (2017)		12 mm	Combined Psy (group and individual therapy: CBT, psychoeducation, combing supportive, interpersonal and exposure techniques, sleep hygiene, relaxation, cognitive restructuring, social support)	105	Asylum seekers	Azerbaijan Burkina Faso Burundi Cameroon Central African Republic of Congo Eritrea Ethiopia Liberia Mali Nepal Pakistan Russia Rwanda Sierra Leone Togo Uganda	PTSD Anxiety Depression Torture	HURIDOCS HTQ HSCL 25	Combined Psy psychotherapy and group therapy, Supportive, psychoeducation, CBT and exposition and reprocessing trauma, weekly	PTSD Depression Anxiety: Significant reduction

*ASI* addiction severity index, *AUDIT* Alcohol use disorder identification test, *BDI* Beck depression inventory, *Beck SSI* scale for suicidal ideation, *BSI* brief symptom inventory, *CAFES* Coffee and family education and support, *CBT* cognitive behavior treatment, *CES-D* Center for Epidemiologic Studies Depression Scale, *CESD-R* Center for Epidemiologic Studies Depression Scale revised, *CGI* clinical global impression, *CIDI* composite international diagnostic interview, *Combined Pha* combined psychological with pharmacological treatments, *Combined Psy* combined psychological treatments, *CPT* cognitive processing therapy, *CR* cognitive restructuring, *CROP* group facilitator for culture sensitive and resources oriented peer, *CSI* clinician structured interview, *CSS* crisis support scale, *CVDTE* checklist of war detention and torture events, *DT* dynamic therapy, *EMDR* eye movement desensitization and reprocessing, *EMDR-R-TEP* eye movement desensitization and reprocessing recent traumatic episode protocol, *EUROHIS-QOL GAF* global assessment of functioning, *h* hours, *HAM-A* Hamilton Anxiety rating scale, *HAM-D* Hamilton depression rating scale, *HRSD* Hamilton rating scale depression, *HSCL (20,25)* Hopkins Symptom Checklist, *HTQ* Harvard Trauma Questionnaire, *HURIDOCS* events standard formats: a tool for documenting human rights violations, *IES* impact of event scale, *IES-R* impact of events scale revised, *IPT* interpersonal psychotherapy, *ISEL* Interpersonal Support Evaluation List, *MEG-ssVEF* magnetoencephalography steady-state visual evoked fields, *MG* monitoring group, *MINI* mini international neuropsychiatric interview, *NET* narratives experiences treatment, *N-FSS* neural flashbacks severity scale, *N-PASS* neural parameters severity scale, *OFSS* orthostatic function severity scale, *OPAFSS* orthostatic parameters flashbacks severity scale, *OPASS* orthostatic parameters severity scale, *PCL* ptsd checklist, *PCL-C* ptsd checklist civilian version, *PDS* posttraumatic stress diagnostic scale, *PSS* physical symptom score, *PSS-SR* physical symptom score self report, *PTGI* posttraumatic growth inventory, *PTSD* posttraumatic stress disorder, *SCID-5* structured clinical interview for DSM.5, *SCL-90* symptom checklist, *SDS* Sheehan Disability Scale, *SF* social functioning, *SF-MPQ* short-form McGill Pain Questionnaire, *SIT* stress inoculation training, *SM* stress management, *SSI*, supplemental security income, *TAU* treatment as usual, *TC* trauma counselling, *TF-CBT* trauma focused cognitive behavioural treatment, *TFT* Thought Field Therapy, *TFT* trauma focalized treatment, *VAS* visual analogue scale, *VCOV* Checklist organized violence, *WB-FACES-PRS 5*, Wong Baker faces pain rating scale, *WHO5* world health organization scale assessment, *WHOQOL(5)* WHO quality of life, *WLC* waiting list control

In the first metanalysis a comparison between pre-treatment and post-treatment values was been performed for each outcome (depression, anxiety, somatization) differentiating the trials based on research design (RCT, MCT, SCT). When two or more measurement scales for the same outcome (for example Hamilton Depression Rating Scale and Beck Depression Inventory) were administered in the same trial, the more frequent scale among all trials included in the meta-analysis was considered. Further sub-analysis, were made dividing the trials according 3 variables: status (refugees, asylum seekers), provenance (Middle East, Africa, Asia, Latin America), and treatment (narrative exposition therapies-NET, cognitive behavioural treatment-CBT, Dynamic therapies, combined psychological treatments, combined pharmacological/psychological treatments). The metanalysis was performed when at least 4 trials were homogeneous for the considered variables.

### Heterogeneity

Evaluation of heterogeneity was reported by the RevMan5.3 program. When the heterogeneity was high (despite the significance of the result), further sub-analysis (status, provenance, single treatment) were made.

### Mean Differences

Pre and post intervention data of all the experimental treatments (within group) were inserted to calculate the means difference. For each trial mean difference, standard deviation, number of subjects at pre and post intervention for one of the three outcomes were provided.

### Metanalytic Techniques Calculation

Mean differences (random effects model) were computed using Review Manager version 5.3. (RevMan5.3). All the mean differences were computed for depression, anxiety and somatization outcome. The program provides the following results: Heterogeneity (Tau^2^, Chi^2^, I^2^) and test for overall effect (Z). Publication bias significance was evaluated by inspecting the funnel plots as implemented in RevMan5.3.

## Results

### Study Selection

**Fig. 1:**
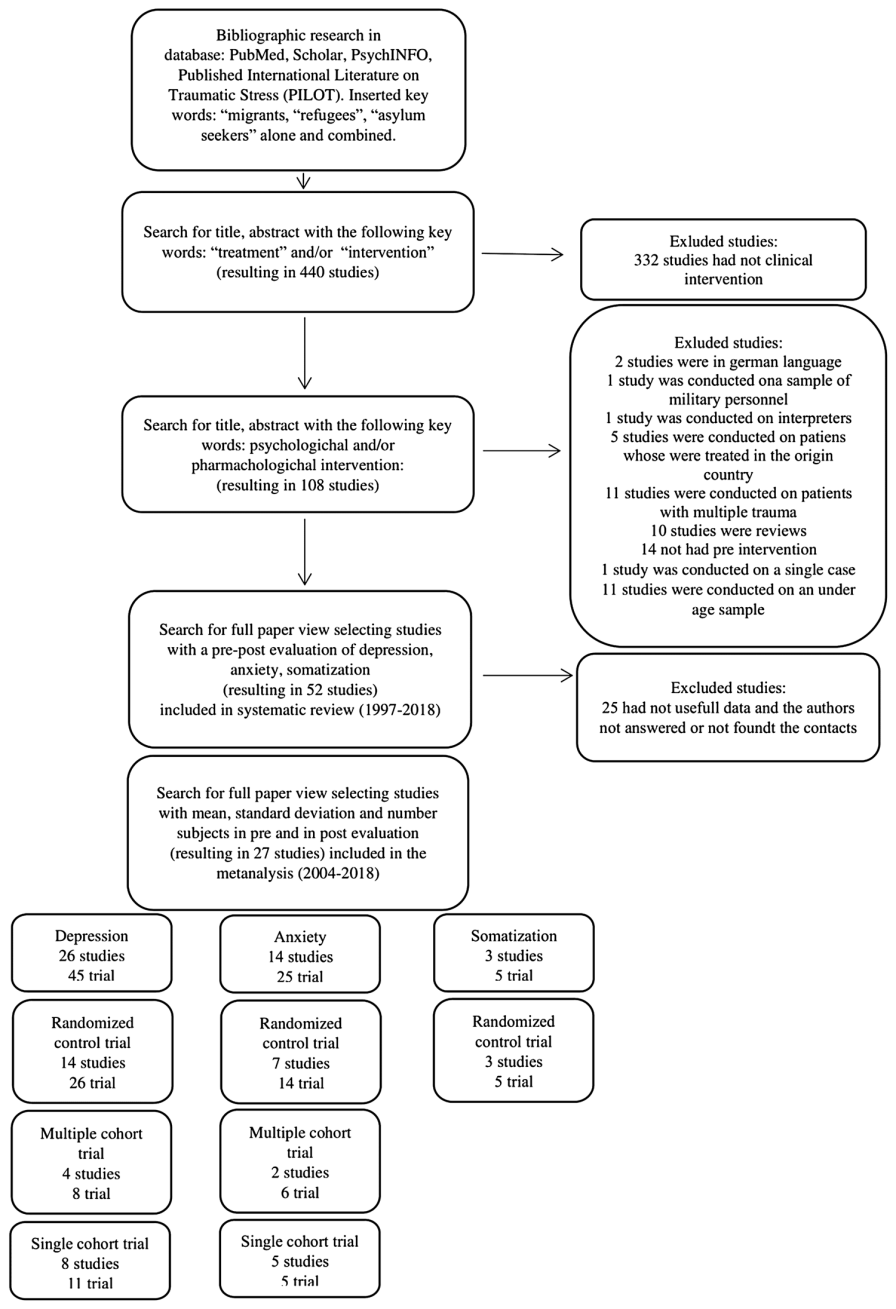
Flow chart selection and organization of the inclusion criteria and the studies for outcome (depression, anxiety, somatization) and research design (randomized controlled trial, multiple cohort trial, single cohort trial)

440 research papers about migrants, refugees or asylum seeker, were included as reported in Fig. 1 (PRISMA). Afterwards the research papers on migrants receiving psychological or/and pharmacological treatment were selected, resulting 108 studies. A more specific full view paper investigation on this subsample of studies, identified only the research paper on migrants with a psychological and/or pharmacological treatment with pre and post evaluation of depression and/or anxiety and/or somatization, resulting 52 studies (1997–2018). These fifty-two studies were included in the systematic review (Supplementary material). Of these 52 studies, 25 were excluded from the metanalysis due to lacking data. Finally, 27 studies (2004–2018) were included for the metanalysis (Review Manager 5.3 program was set for calculating mean differences, standard deviation and number of subjects through a random effects model.

### Excluded Studies

332 on 440 studies were excluded due to the lack of clinical intervention. Of the resulting 108 studies, 56 studies were excluded due to the following reasons: 2 studies were wrote in German language; 1 study was performed on a sample of military personnel; 1 study was conducted on a sample of interpreters; 5 studies were performed on patients who were treated in own country; 11 studies were conducted on patients with multiple trauma; 10 studies were reviews; 14 studies did not present a pre-intervention evaluation; 1 study was conducted on a single case; 11 studies were conducted on an underage sample. Of the resulting 52 studies, 25 studies did not report useful data and the authors did not answer to an email request of data or their email address was not available.

### Systematic Review

Qualitative analysis of the systematic review included 52 studies, 4 studies with control group, 21 studies with a follow up, 21 studies with both follow up and control group, 6 studies without control group and follow up.

Totally, in the 52 studies there were 4720 patients with a pre-treatment evaluation and 3913 with a pre and post treatment evaluations.

Eighty-eight trials, from the 52 studies, provided one of the following treatments: 22 trials performed the CBT (classic CBT treatments, eye movement desensitization reprocessing-EMDR, stress inoculation training-SIT, trauma counselling-TC, thought field therapy-TFT, exposition, stabilization, focalization, cognitive rebuilding-CR, self-management-SM), 9 trials performed the NET (wrote or no wrote), 15 trials performed the dynamic therapies (interpersonal psychotherapy-IPT, group facilitator for culture sensitive and resources oriented peer-CROP, Coffee and family education and support-CAFES, contact and safety planning-CSP, tea and family education and support-TAFES, satisfaction, transcendental meditation-TM, testimony psychotherapy-TP), 20 trials performed the combined cognitive psychological treatment and 23 trials performed the combined pharmacological and psychological treatments.

About legal status category, 31 studies reported refugee samples, 4 studies reported asylum seeker samples, 13 studies reported both refugee and asylum seeker samples and 4 studies reported samples with not specified socio-politic status.

About provenance category, 20 studies treated Middle east populations, 8 studies treated African populations, 7 studies treated Asian populations, one study treated South American populations, 8 studies treated populations of different provenience, 8 studies did not specify the provenance.

Mean number of sessions for each treatment category was respectively: CBT, 13 sessions of 81 min; NET, 7 sessions of 103 min; dynamic therapies, 22 sessions of 105 min; combined psychological treatments, 35 sessions of 59 min; combined psychological with pharmacological treatments, 18 sessions of 62 min.

In the systematic review each sample was considered and included in one, in two or in all the three outcomes (depression, anxiety and somatization) considering the pre-post effect reported in each of the 52 studies resulting in one or two or three trials.

The systematic review included 52 studies for 107 samples (139 trials). Of these 139 trials, 18 trials were excluded because of were not eligible treatments: 16 samples (16 trials) were usual treatments or wait lists and 2 samples (2 trials) were pharmacological treatments. The samples included in the final systematic review were 89 (121 trials). Qualitative results of the systematic review showed that the CBT (22 samples with 28 trials) had significant effect on depression (18 trials) in 11/18 trials (61%) and no significant effect in 7/18 trials (39%). The CBT had significant effect on anxiety (8 trials) in 4/8 trials (50%) and no significant effects in 4/8 trials (50%). The CBT had significant effect on somatization (2 trials) in 0/2 (0%) trials and no significant effect in 2/2 trials (100%).

The NET (9 samples with 9 trails) had significant effects on depression in 4/7 trials (57%) and no significant effects in 3/7 trials (43%). There were not trials using NET on the anxiety outcome. The NET had significant effect on somatization in 1/2 trials (50%) and had not significant effects in 1/2 trials (50%).

The dynamic therapies (15 samples with 16 trials) had significant effect on depression in 5/10 trials (50%) and had no significant effect in 5/10 trials (50%). The dynamic therapies had significant effect on anxiety in 2/4 trials (50%) and had not significant effects in 2/4 trials (50%). The dynamic therapies had significant effect on somatization in 1/2 trials (50%) and had not significant effect in 1/2 trials (50%).

Combined psychological treatments (20 samples with 31 trials) had significant effect on depression in 6/13 trials (46%) and had not significant effect in 7/13 trials (54%). Combined psychological treatments had significant effect on anxiety in 6/13 trials (46%) and had not significant effect in 7/13 trials (54%). Combined psychological treatments had significant effect on somatization in 3/5 trials (60%) and had not significant effect in 2/5 trials (40%).

Combined psychological and pharmacological treatments (23 samples with 37 trials) had significant effect on depression in 8/13 trials (61%) and not significant effect in 5/13 trials (39%). Combined psychological and pharmacological treatment had significant effect on anxiety in 4/11 trials (36%) and had not significant effect in 7/11 trials (64%). Combined psychological and pharmacological treatments had significant effect on somatization in 5/13 trials (39%) and had not significant effect in 8/13 trials (61%).

### Metanalysis

#### Included Studies Characteristics and Outcome

Metanalysis included 27 studies for 75 trials.

15 RCT, 4 of which presented a follow up, 2 showed a control sample, and 9 reported a follow up and control sample.

4 MCT, one of which reported a control sample and 3 showed follow up and control sample.

8 SCT, 6 of which were planned with follow up, and 2 without a control sample and/or a follow up.

Depression outcome was treated in 26 studies (45 trials): 14 studies were RCT with 26 trials, 4 studies were MCT with 8 trials, and 8 studies were SCT with 11 trials.

The anxiety outcome was treated in 14 studies (25 trials): 7 studies were RCT with 14 trials, 2 studies were MCT with 6 trials, and 5 studies were SCT with 5 trials.

The somatization outcome was treated in 3 RCT (5 trials).

Moreover, regarding RCT, 6 studies assessed depression, 6 studies investigated depression and somatization, 1 study examined depression, anxiety and somatization, 1 study assessed depression and somatization, and 1 study considered only somatization. With regard to MCT, 2 studies assessed depression and 2 studies examined depression and anxiety. As regard SCT, 5 studies investigated depression and anxiety and 3 studies considered depression.

As regard the treatment areas for RCT, 7 studies tested CBT treatments, 5 studies analyzed NET, 0 studies assessed dynamic therapies, 1 study investigated combined psychological treatments, and 5 studies examined combined psychological and pharmacological treatments. About treatment area for MCT, 3 studies tested dynamic therapies, and 2 combined psychological treatments. About treatment area for SCT, 1 study assessed CBT treatment, 1 study analyzed NET, 0 studies investigated dynamic therapies, 4 studies examined combined psychological treatments, and 2 studies explored combined psychological and pharmacological treatments.

Moreover, about the provenience area 11 RCT included Middle East populations and 3 studies tested populations from different provenances. About MCT, 3 studies tested Middle East populations, and one study Asian populations. About SCT, 2 studies included Middle East populations, 4 studies tested populations from different provenances, one study assessed African populations, and 2 studies did not specify the provenance of the participants.

Moreover, about the status area, 9 RCT investigated refugees, 2 studies included asylum seekers, 3 studies examined both refugees and asylum seekers, and one study did not specify the status of the migrant participants. As regard MCT, 2 studies included refugees, and 2 studies considered both refugees and asylum seekers. For SCT, 2 studies included refugees, one studies investigated asylum seekers, and 5 studies assessed both refugees and asylum seekers.

### Participants of Metanalysis

The 27 selected studies involved the following number of participants (for all outcome): 956 participants pre-treatment and 774 participants post treatment as regard RCT design; 432 participants pre-treatment and 283 participants post treatment for MCT design; 809 participants pre-treatment and 785 participants post treatment regarding SCT design. The participants differentiated for outcome were: depression 20,177 pre-treatment and 1822 post treatment; anxiety 1225 pre-treatment and 1086 post treatment; somatization 207 pre-treatment and 184 post treatment.

The participants were adult men and women, the age range was 19–51 years for RCT, 18–70 years for MCT, and 18–80 years for SCT.

### Interventions Comparation of Metanalysis

The mean number of sessions was: 9.57 sessions of 98.7 min as regard RCT for CBT; 8.8 sessions of 96.6 min for NET; 10 sessions of 90 min for combined psychological treatments; 11.22 sessions of 70 min for combined pharmacological and psychological treatments. As regard MCT there were found 60 sessions of 24 min for dynamic therapies and 71.5 sessions of 82.5 min for combined psychological treatments. With regard to SCT there were found 9.1 sessions of 60 min for CBT; 3 sessions for NET; 22 sessions of 23.25 min for combined psychological treatments; 47.75 sessions for combined pharmacological and psychological treatments.

### Comparators

Comparators included the experimental samples and control samples if treated with CBT, NET, dynamic therapies with different manualization or combined psychological treatments and combined psychological and pharmacological treatments. There have been excluded the control samples as waiting lists.

### Treatments Effects: Effect Size About Metanalysis

Metanalytic analysis was performed on 27 selected studies separating the studies for outcome, depression, anxiety and somatization and for each different study design (RCT, MCT, and SCT). Successively, because of the high heterogeneity, further sub-metanalysis have been performed following the areas of interest (at least 4 trials were sufficient to perform a metanalysis): socio-politic status (asylum seekers and refugees), provenance, and treatment's kind. The sub-metanalysis were performed considering the different outcomes and the different research designs.

### Depression Outcome

3 main forest plots (7 forest plots, 2 funnel plots inside Supplementary Figures) have been obtained.

**Fig. 2:**
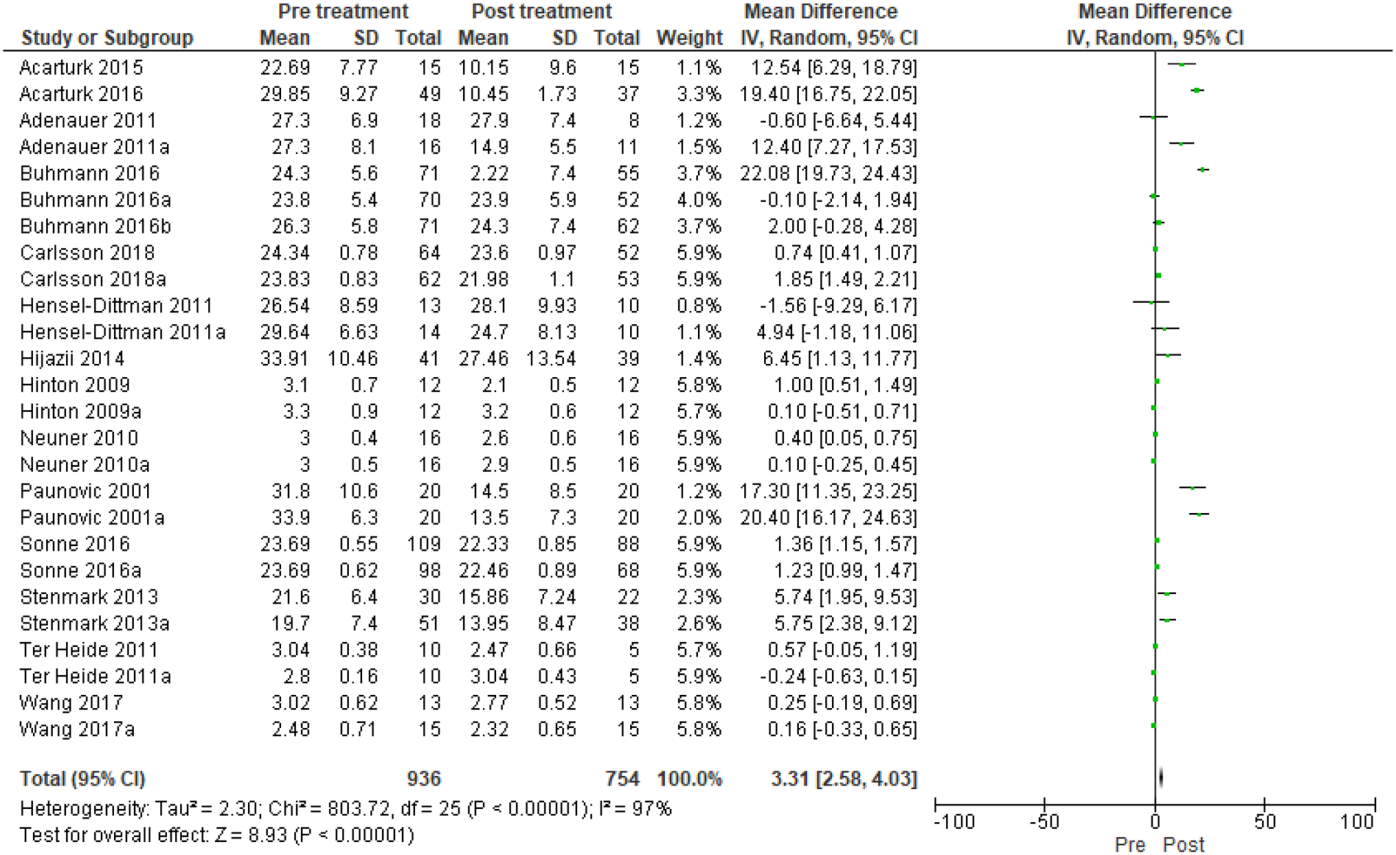
Forest plot that describes the difference pre and post intervention in the depression outcome. RCT research design

RCT-depression (Fig. 2): 936–754 participants pre-post treatment; Heterogeneity: Tau^2^ 2.3, Chi^2^ 803.72, df 25 (p < 0.00001), I^2^ 97%; test for overall effect: Z 8.93 (p < 0.00001).

MCT-depression: 432–283 participants pre-post treatment; heterogeneity: Tau^2^ 0.08, Chi^2^ 25.02, df 7 (p = 0.0008), I^2^ 72%; test for overall effect: Z 4.08 (p < 0.0001).

SCT-depression: 809–785 participants pre-post treatment; Heterogeneity: Tau^2^ 0.56, Chi^2^ 269.25, df 10 (p < 0.00001), I^2^ 96%; test for overall effect: Z 6.45 (p < 0.00001).

### Sub-metanalysis Results on the Kind of Treatment for Depression RCT

CBT: 213–162 participants pre-post treatment (7 studies—8 trial). Heterogeneity: Tau^2^ 5.15; Chi^2^ 230.88, df = 7, p < 0.00001; I^2^ 97%; test for overall effect: Z 4.02 p = 0.0001.

NET: 138–114 participants pre-post treatment (5 studies—5 trial). Heterogeneity: Tau^2^ 23.22; Chi^2^ 37.02 df = 4 p < 0.00001; I^2^ 89%; test for overall effect: Z 2.4 p = 0.02.

Combined psychological and pharmacological treatments: 539–442 participants pre-post treatment (5 studies—10 trial). Heterogeneity: Tau^2^ 2.30; Chi^2^ 444.14 df = 9 p < 0.00001; I^2^ 98%; test for overall effect: Z 7.44 p < 0.00001.

### Sub-metanalysis Results on the Kind of Status for Depression RCT

Refugees: 714–585 participants pre-post treatment (8 studies—14 trial). Heterogeneity: Tau^2^ 3.28; Chi^2^ 640.06 df = 13 p < 0.00001; I^2^ 98%; Test for overall effect: Z 9.34 p < 0.00001.

Asylum seekers: 87–80 participants pre-post treatment (3 studies—6 trial). Heterogeneity: Tau^2^ 0.00; Chi^2^ 3.99 df = 5 p < 0.55; I^2^ 0%; test for overall effect: Z 2.36 p < 0.02.

### Sub-metanalysis Results for Kind of Status for Depression SCT

Refugees: 327–312 participants pre-post treatment (4 studies—5 trial). Heterogeneity: Tau^2^ 1.40; Chi^2^ 143.98 df = 4 p < 0.00001; I^2^ 97%; Test for overall effect: Z 4.03 p < 0.0001.

### Sub-metanalysis Results on the Kind of Provenance for Depression RCT

Middle East: 837–662 participants pre-post treatment (11 studies—20 trial). Heterogeneity: Tau^2^ 2.34; Chi^2^ 654.76 df = 19 p < 0.00001; I^2^ 97%; test for overall effect: Z 7.75 p < 0.00001.

### Sub-metanalysis Results for Kind of Treatment for Depression SCT

Combined psychological treatment: 313–295 participants pre-post treatment (4 studies—4 trial). Heterogeneity: Tau^2^ 0.04; Chi^2^ 15.59 df = 3 p = 0.001; I^2^ 81%; Test for overall effect: Z 4.08 p < 0.0001.

### Anxiety Outcome

**Fig. 3:**
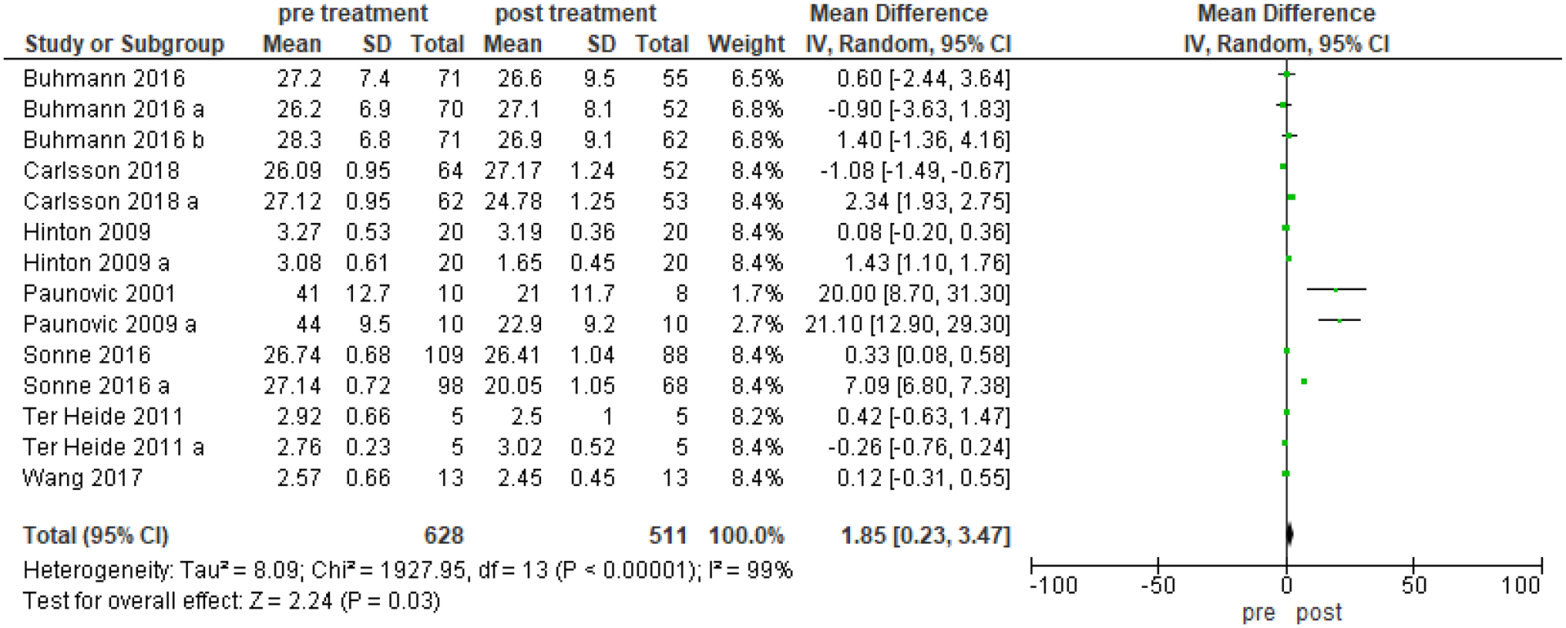
Forest plot that describes the difference pre and post intervention in the anxiety outcome. RCT research design

RCT-anxiety (Fig. 3): 628–511 participants pre-post treatment; heterogeneity: Tau^2^ 8.09, Chi^2^ 1927.95 df 13 (p < 0.00001), I^2^ 99%; test for overall effect: Z 2.24 (p = 0.03).

MCT-anxiety: 108 participants pre e post treatment; heterogeneity: Tau^2^ 0.49, Chi^2^ 91.99, df 5 (p < 0.00001), I^2^ 95%; test for overall effect: Z 2.69 (p = 0.007).

SCT-anxiety: 489–467 participants pre-post treatment; heterogeneity: Tau^2^ 0.06, Chi^2^ 28.66 df 4 (p = 0.00001), I^2^ 86%; test for overall effect Z 3.40 (p = 0.0007).

### Sub-metanalysis for Kind of Status on Anxiety RCT

Refugees: 605–488 participants pre-post treatment (5 studies—11 trial). Heterogeneity: Tau^2^ 9.31; Chi^2^ 1813.09 df = 10 p < 0.00001; I^2^ 99%; test for overall effect: Z 2.49 p = 0.01.

### Sub-metanalysis for Kind of Provenance on Anxiety RCT

Middle East: 558–443 participants pre-post treatment (4 studies—8 trial). Heterogeneity: Tau^2^ 12.49; Chi^2^ 1664.77 df = 7 p < 0.00001; I^2^ 100%; test for overall effect: Z 1 p = 0.32.

### Sub-metanalysis for Kind of Treatment on Anxiety RCT

Combined psychological with pharmacological treatments: 535–436 participants pre-post treatment (5 studies—10 trial). Heterogeneity: Tau^2^ 9.32; Chi^2^ 1809.25 df = 9 p < 0.0001; I^2^ 100%; test for overall effect: Z 2.69 p = 0.007.

### Somatization Outcome

**Fig. 4:**
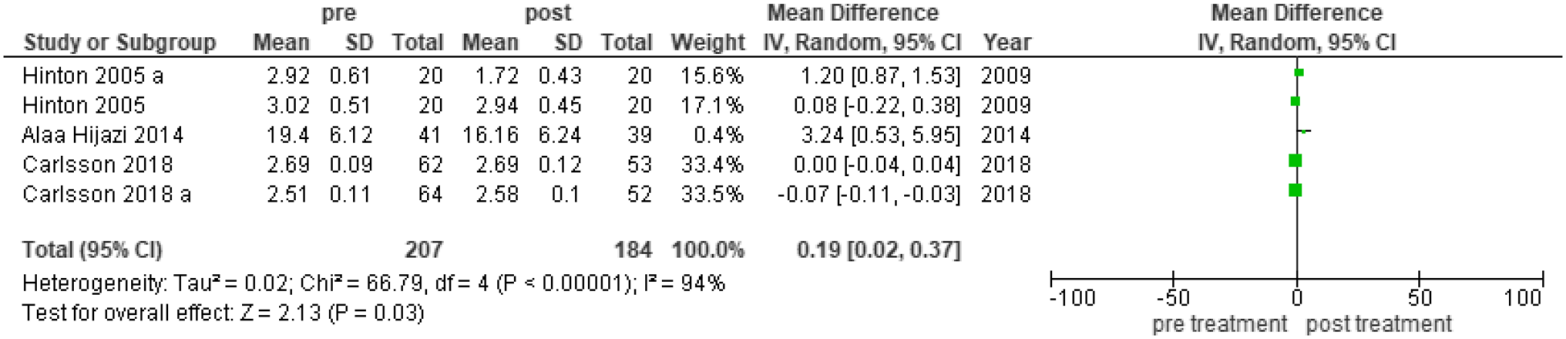
Forest plot that describes the difference pre and post intervention in the somatization outcome. RCT research design

RCT-somatization (Fig. 4): 207–184 participants pre-post treatment. Heterogeneity: Tau^2^ 0.02, Chi^2^ 66.79, df 4 (p = 0.00001), I^2^ 94%; test for overall effect: Z 2.13 (p = 0.03).

### Methodological Quality: Publication Bias

The risk of bias among studies was high for many reasons, 13/27 (metanalysis) 30/52 (systematic review) publications were MCT and SCT studies this was the first issue hampering the methodological quality of the studies. Moreover, in many studies the number of participants at the pre-treatment phase (T0) decreased in the post treatment (T1). Often treatments with the same approach were manualized in different ways. In some studies, the samples included both refugees and asylum seekers. The different status kind could have a different impact on the psychological health of the participants. Many studies did not have an appropriate random generation assignment sequence and did not report an occultation of the assignment sequence. Some trials coming from different studies, sometimes, used different instruments of measure to evaluate the same constructs.

Further, different types of bias were detected in the studies selected for the present metanalysis and included: bias of data base, bias of inclusion, bias of language, and bias of effect size. Moreover, some studies did not report possible risks of bias. The summary of all biases and the high heterogeneity were reported in two funnel plots. However, the two funnel plots showed a good symmetry.

## Discussion

The main finding of the present metanalysis is that all treatments categories, and particularly the cognitive behavioural treatments, showed to be efficient on depression.

Moreover, the effect of the treatment was lower on the asylum seekers compared to the refugees.

Specifically, the cognitive behavioural treatments together combined psychological and pharmacological treatments seemed to be more efficacious on depression, even if in a lower number of studies.

The dynamic therapies showed a discrete efficacy trough observational methodological designs, there is a need of further investigation in this field due to the total lack of RCT.

As regard the migrant’s status (refugee or asylum seeker), the efficacy of the treatment on the depression showed a significant effect only for the status of refugee, whereas the effect was at the limit of significance with null degree of heterogeneity for the state of asylum seeker. This difference be could be due to adherence to the institutional expectation common in the asylum seekers applicants. In facts, they may have distorted the answers to the questionnaires in order to support or to influence the acceptation of their instance to receive an international protection [40]. This social desirability bias could modulate the pre-post evaluation differences or to attenuate the efficacy of the psychological intervention. This finding suggests to consider with great attention the variable of the status, and overall the pending instance condition of the migrant participants in the future studies. Considering that UNHCR data in Italy reported that in the first half of 2017 the total number of applications examined amounted to 41,379; only 4.3 out of 10 had a positive outcome (refugee status: 9%; subsidiary protection: 9.8%; permit for humanitarian reasons: 24.5%); for 51.7% the exam ended with a denial, and 4.9% of the instances of the applicants were lost [41].

Other important results were that the psychological interventions showed an effect on the mental health of migrants. The more evident effect of the psychological treatments was on the depression, despite the systematic review showed that only the 50% of the RCT found a significant difference between the pre and post evaluation. The specific metanalysis and sub-metanalysis showed high significance levels. For all the types of treatments with the exception of the narrative exposure therapies, that showed effects at the limit of significance. This finding suggest to conduct further experimental studies in order to confirm the efficacy of narrative exposure therapies on the depression outcome. Moreover the combined (psychological and pharmacological/psychological) treatments seem to show increased benefits compared to the single ones.

The metanalysis on depression in not randomized studies (multiple and single perspective design studies) showed high levels of significance but with high levels of the heterogeneity.

Differently from the depression, the anxiety showed smaller effects of the psychological treatment. Not only the metanalysis showed decreased pre-post treatment effects on anxiety, but also the qualitative observation on the systematic review showed a probability lower than 50% to have a significant pre-post treatment effects on trials with anxiety. Moreover, the number of the studies criteria allowed to perform only the metanalysis on the RCT studies with the combined psychological/pharmacological treatments where the effect was statistically significant. It seems necessary to plane further randomized studies in order to test the efficacy of specific psychological treatments on outcomes other than depression.

Moreover, the metanalysis performed only in middle-east migrant population showed a clear statistical pre-post effect of the psychological treatment on the depression outcome but not on the anxiety outcome. This result could be explained with the cultural characteristics of these populations where the expression of the anxiety is under-represented compared to other cultures [42].

Another important finding of the present study was that, at today, there are few studies on African and Asian populations compared to the Middle East population. In the last years, a large entrance in Europe of the African population, as well as Syrian and Iranian population, has been observed. It seems to be necessary to increase the trials on the participants who have been involved in the current phenomena of migration as African, Asian, Syrian, and Iranian populations.

As regard the somatization outcome, the qualitative observation from the systematic review showed a probability of 42% to have a significant pre-post treatment effects on randomized control, multiple and single perspective cohort trials of 42%. This limited qualitative evidence was confirmed by the metanalyses on the RCT where the statistical effect was at the limit of significance. This finding suggests the need to conduct more studies focused on testing the efficacy of psychological treatments on somatization, talking into consideration of the population at today involved in the migration phenomena characterized by a culture where somatization could represent a privileged channel to express their psychological disease [43].

Moreover, the limited number of studies with outcome of anxiety and somatization did not allow to compare the efficacy of different types of psychological treatment on these outcomes revealing a hole in the literature that it is necessary fil up in the next future.

## Future Research

For future developments, we suggest to extend the research to the outcome of psychological support for the migrants using a dynamic approach, possibly through RCT design. Moreover, it seems to necessary to confirm the efficacy of narrative exposure therapy on the depression increasing the studies number on this issue. According to this line, it could be interesting to turn the attention also to the expressive writing treatment of Pennebaker [44]. This treatment shows is more systematized procedure compared to the other narrative exposure therapies and it seems to demonstrate a good efficacy in brief times (3–5 days) adopting an exclusively written application modality. Nowadays, this type of treatment was not tested on migrants yet.

Finally, it would be useful increases the number of studies of all psychological treatments applied on anxiety and somatization, possibly through RCT design.

## Limitations

Few studies among those included in this systematic review treated the asylum seekers respect to the refugees.

## Electronic supplementary material

Below is the link to the electronic supplementary material.

Supplementary file 1 (DOC 819 kb)
