# Supplementary material for: Efficacy of Psychological Interventions on Depression Anxiety and Somatization in Migrants: A Meta-analysis
Source: J Immigr Minor Health. 2020 Jul 25;22(6):1320–46. doi: 10.1007/s10903-020-01055-w (PMC7683473; doi:10.1007/s10903-020-01055-w)
Supplement: Supplementary file 1 — (DOC 819 kb) [file 10903_2020_1055_MOESM1_ESM.doc]

Table 1s. Description of treatment studies examining the efficacy in traumatized migrants adult. 52 studies are included: 27 for the metanalysis and 25 considered in the a systematic review

| Refs. | RCT | Control | Follow up | Samples | Subjects  (total and for experimental samples pre and post. In order of samples) | Status | Provenience | Outcome | Measures | Experimental samples  CBT treatments-NET-Psychodinamic therapy-Combined Psychological treatment (Combined psy)-Combined Pharmachological and psychological treatment (Combined pha) | Findings for each treatment applied outcome (the outcomes were not reported, they were no significance) |
| --- | --- | --- | --- | --- | --- | --- | --- | --- | --- | --- | --- |
| [13] | Acarturk 2015 | Yes | 1 mm | -CBT (EMDR)  -WCL( no treatment) | 29  15  14 | Refugees | Siria | TEI  PTSD  Depression | IES-R  BDI II | -CBT (EMDR)  7 sessions, 90 minutes, weekly. | PTSD  Depression:  significantly lower. remission mainteined |
| [14] | Acarturk 2016 | Yes | 1 mm | -CBT (EMDR Focus recent trauma prolonged at present time)  -WCL (no treatment) | 98  49/37  49/33 | Refugees | Siria | TEI  PTSD  Depression  Neuropsychiatric symptomatology | HTQ  IES-R  BDI-II  HSCL 25  MINI | -CBT (EMDR)  4 sessions | PTSD  Depression  Impact event  significantly symptoms reduced, after 1 month mainteined |
| [15] | Adenauer 2011 | Yes | 4 mm | -NET (Writed by therapists and reading high voice to subject)  -WLC (no treatment or antidepressants) | 34  16/11  18/8 | Refugees  Asylum seekers |  | PTSD  Depression  Dystimya  Neurocorrelates | MEG-ssVEF  CSI  CWDTE  CAPS  HAM-D  MINI | -NET  12 sessions, 108 minutes, weekly or biweekly | PTSD  Depression significant effectiveness significant Changing of the neural correlates of the processing. NET increased occipital and parietal activity After 4 mm. maintained |
| [16] | Buhmann 2016 | Yes | 2 mm  4 mm  6 mm.  8mm. | -CBT (commitment therapy and mindfulness andvisual esposition)  -Combined Psy (CBT and psychoeducation and antidepressants  -Combined Pha (psychoeducation and sertraline or mianserine  -WLC (no treatment) | 280  70/52  71/62  71/55  68 | Refugees | Iraq  Iran  Lebanon  Yugoslavia  Afghanistan | PTSD  Anxiety  Depression  Somatization  Stress  Panic  InabilityPsychofisical | HTQ  HSCL25 SCL90  HAM-D  HAM-A  VAS  SDS  WHO-5 | -CBT  16 sessions, weekley  -Combined psy  4 sessions, weekley  -Combined pha 8 sessions, weekley | Depression: treatment with antidepressant in combination psychoeducation was associated with significant decrease and sygnificantly decrease in patients reciving medicine.  Anxiety: significant effect of medication |
| [17] | Carlsson 2018 | Yes |  | -Combined Pha (CBT SM for acquire coping skills, relax, divided attention, behavioural activation) and pharmachological (mianserin or sertraline) and psychoterapy  -Combined Pha (CBT CR psicoeducation, cognitive rebuilding of negative ideas.) and pharmachological (mianserin or sertraline) and psychoterapy  -TAU | 140  62/53  64/52 | Refugees | Afghanistan  Yugoslavia  Iran Iraq Lebanon | PTSD  Panic,  Anxiety  Depression  Somatization  Wellbeing  Disability,  Functioning;  Symptomatology | HTQ  HSCL 25  HAM D  HAM A  SCL  VAS  WHO  SDS  GAF S  GAF F | -Combined Pha  (SM and CR)    24 sessions, and 10 sessions with medical doctor, 16 sessions with psychoterapist. 60 minutes beweekley | PTSD  Somatization  Functioning  Disability:  CR significant effect.  Depression  Anxiety  Somatization:  SM significant effects  Anxiety: beta significant |
| [18] | Hensel-Dittmann 2011 |  | 4 ww  6 mm  12 mm | -NET (Required by therapysts and corrected by subject with reading)  -CBT (SIT: cognitive behaviour semistructured intervention) | 28  14/10  13/10 | Asylum seekers |  | PTSD  Depression  Neurosymptomatology | CAPS  HAM-D  MINI | -NET  10 sessions, 90 minutes  -CBT (SIT)  10 sessions | PTSD Sygnificatively reduction with NET.  Maintained at 6 and 12 mm |
| [19] | Hijazi Alaa 2014 | Yes | 2 mm  4 mm | -brief NET (Write the therapists)  -WLC (no treatment) | 63  41/39  22 | Refugees | Iraq | PTSD  Depression Postraumatic growth  Psychological wellbeing  Symptomatology of stress postraumatic  Somatization | HTQ  PTGI  WHO5  BDI-II  PHQ-15 | -Brief NET  3 sessions, 60-90 minutes, weekley | PTSD  Depression  Somatization:  Reduction at 2 and 4 mm.  Well being improved at 2 and 4 mm |
| [20] | Hinton 2004 |  | 22 ww | -Combined Pha (Immediate CBT and SSRI, benzodiazepine, gabapentin)  -Combined Pha (Delayed CBT and SSRI, benzodiazepine, gabapentin) | 24  12  12 | Refugees | Vietnam | PTSD  Depression  Anxiety  Panic  Psychophisical symptomatology | HTQ  ASI  HSCL 25  HPASS  OPASS | -Combined Pha immediate CBT 11 sessions  -Combined Pha delayed CBT started after 11 sessions for another 11 sessions | PTSD  Depression  Anxiety:  Imrovement since 1 assessment to 2 assessment for immediate and retand treatments. Assessment since 1 to 3 significant improvement  Psychophisical symptomatology, improve significantly |
| [21] | Hinton 2005 |  | 28 ww | -Combined Pha(immediate CBT: and SSRI, Clonazepam, social support)  -Combined Pha (delayed CBT: and SSRI, Conazepam, social support) | 40  20  20 | Refugees | Cambogia | PTSD  Anxiety  Somatization | ASI  CAPS  N-PASS  O-PASS  N-FSS  O-FSS  SCL90R | -Combined Pha immediate CBT 12 sessions  -Combined Pha delayed CBT after 12 sessions for another 12 sessions | PTSD  Anxiety  Panic  Orthostatic parameters  Flashbacks:  Significant group effect, time effect and interaction.  Follow up:  Significant time effect and group effect and time interaction.  On all measures Immediate group has significant lower scores at second assessment. Delayed CBT improvement at 3 assessment |
| [22] | Hinton 2009 |  | 24 ww | -Combined Pha(immediate CBT: and paroxetine, Clonazepam, social support)  -Combined Pha (delayed CBT: and Paroxetine, Clonazepam, social support) | 24  12  12 | Refugees | Cambogia | PTSD  Emotional regulation  Orthostatic parameters  Catastrophic cognition  Flashbacks | CAPS  ERS  OPAI  OPASS  OPAFSS | -Combined Pha immediate CBT 12 sessions  -Combined Pha delayed CBT after 12 sessions for 12 sessions | PTSD  Somatization  Flashbacks:  significant improvement at 2 assessment.  PTSD: CBT decreased pain and increased emotional regulation and vagal tone that improvemented the blood pressure |
| [23] | Meffert 2014 | Yes | Since 3 to 6 mm | -CBT: (IPT, group treatment)  -Control: CBT (IPT) | 19  11  8 | Refugees | Sudan | PTSD  Depression  Conflict  Anger | HTQ  BDI  CTS  STAXI | -CBT (IPT)  12-16 sessions, beweekley | PTSD  State anger  Depression  CBT (IPT) predicted a significant decrease |
| [24] | Neuner 2004 |  | 4 mm  12 mm | -NET (Writing by therapist  and reading together)  -CBT (counselling)  -Dynamic (psychoeducation) | 43  17/15  14/13  12 | Refugees | Sudan  Uganda | PTSD  Distress  Suicide Generic health | DFMQ  PDS  SRQ20  SF12  CIDI | -NET 4 sessions, 90-120 minutes, beweekley  -CBT counselling  4 sessions  -Dynamic psychoeducations  1 session | PTSD: 12 mm follow up NET significant improvement respect CBT and Dynamic. The difference there was on all outcome significant post treatment |
| [25] | Neuner 2008 | Yes | 3 mm  6 mm  9 mm | -NET (write by therapists and readed)  -CBT (TC)  -MG | 277  111  111  55 | Refugees | Ruanda Somalia | Symptomatology on stress postraumatic  Physichal symptoms | CIDI  PDS  PSS | -NET and CBT (TC)  6 sessions, beweekley | PTSD  Physic health  Both treatments are superior MG on PTSD and physic healt but nobody significatively differences at follow up. CBT and NET significatley more efficacy respect MG on each measure but not to follow up. Between NET and CBT no significant difference. |
| [26] | Neuner 2010 | Yes | 6 mm | -NET (more of 8000 words with supervision)  -CBT (Stabilization or psychoactive medication and TFT) | 32  16  16 |  | Turkey  Balkans  Africa | Depression  PTSD  Pain | PDS  CIDI  HSCL25  CAPS  VCOV | -NET and CBT (Stabilization and TFT)  9 sessions, 120 minutes, weekly and biweekly | PTSD:  Significant main effect time and time for treatment interaction. NET significant difference within and between on PTSD, also to 6 mm.  Pain::  Significant time for treatment interaction |
| [27] | Nordbrandt 2015 | Yes | 12 mm | -Combined Pha (CBT, basic body awareness therapy, physical activity and medical treatment)  -Combined Pha (CBT, mixed physical activity, medical treatment)  -Combined Pha TAU (manual based CBT and medical treatment) | 310 | Asylum seekers | Iraq Palestina Afghanistan Iran  Bosnia  serbia | PTSD  Depression  Anxiety  Quality life  Functional capacity  Pain  Body awareness  Physical fitness | HAM-D  HAM-A  GAF  GAS | -Combined Pha  CBT 16 sessions, 60 minutes  Physical activity  20 sessions 60 minutes.  6-7 mm medical treatments |  |
| [28] | Paunovic 2001 |  | 6 mm | -Combined Pha (CBT: Exposition and tricyclics and SSRIs) or bensodiazepines or neuroleptics or muscle relaxants or neuroleptic)  -Combined Pha (CBT: exposition and cognitive therapy and control breathing and tricyclics and SSRIs or bensodiazepines or neuroleptics or muscle relaxants or neuroleptic) | 40  20  20 | Refugees |  | PTSD  Depression  Anxiety  Event impact  Quality life | CAPS IV  HAM D  HAM A  PSS-SR  IES R  BDI  STAI-S  QOLI  WAS  BAI  ADIS | -Combined Pha  CBT Exposition, 8 sessions, 20-60 minutes, weekley  CBT, 16-20 sessions, 60-120 minutes, weekley | PTSD  Depression  Anxiety  Event impact  Quality life:  CBT significant, better at post test. Both efficacy within on all measure and maintained at follow up |
| [29] | Renner 2011 | Yes | 3 mm  6 mm | -CBT Female (progressive relaxation, breathing technique)  -CBT Male  -CBT (EMDR) F/M  -Dynamic Female  (CROP)  -Dynamic Male (CROP)  -Control (dynamic female CROP)  -control (dynamic male CROP) | 94  10/5  11/5  17/6  10/3  15/9  15/7  16/9 | Refugees Asylum seekers |  | PTSD  Depression  Anxiety | HTQ  HSCL-25 | -CBT (M-F)  15 sessions, 90 minutes, weekley,  -CBT (EMDR)  3 sessions  -Dynamic (CROP,  M-F)  15 sessions, 90 minutes, weekly.  -Control (Dynamic CROP delayed between 4-8 mm M-F)  15 sessions, 90 minutes, weekley | PTSD:  Dynamic significantly superior Control Dynamic at post treatment. Dynamic significantly superior EMDR at T4. Control Dynamic significantly decrease T2-T3.  Depression and Anxiety:  CBT and Dynamic significantly superior to EMDR and Control Dynamic |
| [30] | Sonne 2016 | yes |  | -Combined Pha (CBT and manualized psychotherapy and social counselling and mindfullness and Sertraline max 75 mg)  -Combined Pha (CBT and manualized psychotherapy and social counselling and mindfullnessand  Venlafaxine max 50 mg)  -Pharmacological treatment (same Sertraline) | 207  108/88  98/68 | Refugees | Middle East | CPTSD  Depression  Anxiety  Smatization | HTQ  HAM D  HAM A  HSCL 25  SCL 90  SDS  GAF  GAS  VAS  CSS  WHO5 | -Combined Pha  24 sessions,  10 sessions medical doctor, 16 sessions psychologist | CPTSD  Depression  Anxiety:  Good results for SSRI.  Functioning:  significant difference within for Sertraline and significant differences between for both.  SAS significant differences both within.  GAF S significant difference between |
| [31] | Stenmark 2013 | Yes | 1 mm  6 mm | -NET (no written)  -CBT (Focalization and help for psychological problem) | 81  51/38  30/22 | Refugees Asylum seekers | Middle East | PTSD  Depression  Neurologichal state | CAPS  HAM D  MINI | -NET: 10 sessions, 90 minutes.  -CBT (Focalization): 10 sessions 86 minutes | PTSD  Depression:  Both refugees and asylum seekers reduced their mental problems. Both treatment symptomatologichal rediction but more pronounced for NET. Between group at 6 mm no significant effects. CAPS and HAM D significant main effects of time |
| [32] | Ter Heide 2011 | Yes | 3 mm | -CBT (Stabilization: here and now on trauma memory)  -CBT (EMDR) | 20  10/5  10/5 | Refugees Asylum Seekers | Afghanistan  Algeria  Bosnia  Turchia  Angola  Lebanon | PTSD  Depression  Anxiety  Quality life  Neuropsychiatric state | HTQ  HSCL 25  WHOQOL brief  SCDI 1  MINI 10 | -CBT (Stabilization and EMDR)  11 sessions, weekly and beweekly | PTSD: significant difference between conditions, EMDR some improvement. Significant differences between treatments for HSCL-25 (anxiety and depression) and WHOQOL, EMDR improvements. |
| [33] | Wang 2017 | Yes | 3 mm  6 mm | .Cobined Psy immediate (CBT psychoterapy, grouptherapy and multivitaminic)  -Combined Psy delayed: control (CBT psychoterapy, grouptherapy delayed after 3 mm and multivitaminic immediate) | 34  13  15 | Asilum seekers | Kosovo | PTSD  Depression  Anxiety  Panic | HTQ  HSCL 25  SF.MPQ  WB FACES  WHODAS | -Combined Psy  CBT 10 sessions, 90 minutes,  group therapy 10 session, 90 minutes weekly | PTSD: Significant the effect of intervention at 6 mm.  Pain: at 3 mm. |
| [34] | Weine 2008 | Yes | 6 mm  12 mm  18 mm | -Dynamic (CAFES: 5 phases, 1 joining engagement (1 session), defining the family (3 sessions), working together in the family (3 sessions), using resources outside of the family (2 sessions))  -Control (no intervention, longitudinal assessment) | 197  110  87 | Refugees | Bosnia-Herzegovina | PTSD  Depression | CAFES  PTSD-SS  CFESDS | -Dynamic (CAFES)  9 sessions | Depression: multiple-family group was effective in increasing access to mental health services and depression and family comfort.Significant effect knowlwledge and family comfort |
|  | Multiple prospective cohort trials |  |  |  |  |  |  |  |  |  |  |
| [35] | Drozdek 2010 | Yes | 6 mm  12 mm | -Combined Psy 3*3  -Combined Psy 3*2  -Combined Psy 2*2  -Dynamic: 1*1  -Control (Pharmachological)  5 phases:  1 norms, values of group treatment, psychoeducation, alliance, assessment of problems, treatment goals and symptoms.  2 presentations, damage core beliefs, fear of loss control, guilt, shame, grief, acknowledgement, resilience.  3 telling the trauma story, exposure and cognitive restructuring  4 reconnecting the present with past and future, damage core beliefs, roles and identity, coping strategies, current worries and future outlook, resilience.  5 psychoeducation, relapse prevention, treatment evaluation, farewell ritual. (employed dinamic and behavior tecniques) | 88  34  19  11  6  18 | Refugees Asylum seekers | Iraq  Iran  Afghanistan | PTSD  Depression  Anxiety  Stress  Psychofisic symptoms  Psychotic symptoms | HTQ  HSCL-20  SLC-90 Psychoticism scale | Specifically:  Group therapy 58 sessions, 90 minutes, daily  No verbal therapy  58 sessions, 75 minutes, daily  -Combined Psy 3*3  No verbal psychotherapy, 3 sessions (psychomotor body therapy, art therapy, music therapy) Group therapy, 2 sessions, 3 days week  -Combined Psy 3*2 same thing of 3*3 but 2 days week)  -Combined Psy 2*2 psychotherapy 2 sessions  Group therapy 1 sessions, 2 days week  -Dynamic 1*1  Support, 48 sessions, weekly | PTSD  Depression  Anxiety  Psychoticism  Combined 3*3 and 3*2 Significant effect within  PTSD ( 2*2).  Differences between not statistically significant |
| [36] | Drozdek 2012 | Yes | 12 mm | -Combined Psy 3*3  -Combined Psy 3*2  -Combined Psy 2*2  -WCL (for 6 mm no treatment)  5 phases:  1 norms, values of group treatment, psychoeducation, alliance, assessment of problems, treatment goals and symptoms.  2 presentations, damage core beliefs, fear of loss control, guilt, shame, grief, acknowledgement, resilience.  3 telling the trauma story, exposure and cognitive restructuring  4 reconnecting the present with past and future, damage core beliefs, roles and identity, coping strategies, current worries and future outlook, resilience.  5 psychoeducation, relapse prevention, treatment evaluation, farewell ritual. (employed dinamic and behavior tecniques) | 71  27  22  7  16 | Refugees Asylum seekers | Iran, Afghaniztan | PTSD  Depression  Anxiety  Stress  Psychophysic symptoms | HTQ  HSCL20  SLC90 | Specifically:  Group therapy 58 sessions, 90 minutes, daily  No verbal therapy  58 sessions, 75 minutes, daily  -Combined Psy 3*3  No verbal psychotherapy, 3 sessions (psychomotor body therapy, art therapy, music therapy) Group therapy, 2 sessions, 3 days week  -Combined Psy 3*2 same thing of 3*3 but 2 days week)  -Combined Psy 2*2 psychotherapy 2 sessions  Group therapy 1 sessions, 2 days week | PTSD  Depression  Anxiety  Stress  Psychophysics simptoms  The treatments were significant but 2*2 not significant effect on PTSD.  No significant difference between treatments and treatments and control, but 3*3 and 3*2 more efficacy of 2*2. |
| [37] | Kruse 2009 | Yes | 12 mm | -Combined Psy (CBT and dynamic. 7 phases, development of the treatment relationship, development of a feeling of safety, psychoeducation, cognitive restructuring, muscle relaxation, skill building and regulation affect, self perception and caring for oneself)  -TAU (no specified) | 70  35/34  35/30 | Refugees | Bosnia | PTSD  Somatization  GSI | HTQ  SCI 90  SF 36 | -Combined Psy  30 sessions, 120 minutes, weekly and beweekley | PTSD, significant effect at 12 mm  GSI significant effect at 12 mm  Somatization improved |
| [38] | Lakshmi Vijayakumar 2017 | Yes | 6 mm  15 mm | -Dynamic (CASP: emotional support ontact with a volontary community and support of the planning cards at moment of distress. Medical doctor)  -Control  (to provide the telephon number that can use for supportive help) | 485  288/139  187 | Refugees | Tamil Nadu (India) | Depression  Suicide ideation  PTSD  Alcohol abuse | Beck  SSI (in WHO)  SUPRE MISS  CESD-R  AUDIT  PCL | -Dynamic (CASP)  60 sessions, 4 mm medical doctor | PTSD and Depression, significant differences beetween on, and reduce of 2 unites the means of the scores (signignificant within) |
| [39] | Rees 2013 | Yes | 1 mm  135 dd | -Dynamic (TM: introductory lectures,, personal instructions, follow up, optional group meeting)  -WLC (delayed start) | 42  21  21 | Refugees | Congo | PTSD | PCL-C | -Dynamic (TM)  60 sessions, 270 minutes, twice daily, morning, afternoon,  group meeting, weekly | PTSD: TM group significant reduction PCL scores. After 135 gg maintained results |
| [40] | Schaal 2009 |  | 3 mm  6 mm | -NET (3 NET condition and 1 ITP. Constructed detailed own byography)  -Dynamic (IPT: interpersonal psychotherapy) | 26  12  14 |  | Ruanda | PTSD  Depression | CAPS  MINI  HAM-D | -NET  4 sessions, 120-150 minutes, weekly  -Dynamic (IPT)  4 sessions, 120-150, weekly | PTSD: the differences between the condition treatments at follow up was significant. NET larger effect size on PTSD and depression and major depressive episode.. There were not significant significant differences on depression and major depressive episode. The partecipants in both conditions showed a significant changes in |
| [41] | Weine et al 2003 | Yes | 3 mm | -Dynamic (TAFES multifamily group intervention Home visiting + 6 meetings group family)  -Control | 86  73  13 | Refugees | Kosovo | PTSD  Social sources  Vulnerability of population  Family processes  Service use  Knowledge trauma mental health | TAFES  FPSC  FHI  ISEL  Check list of service use  25 item questionnaire |  | Social support, psychiatric contact, attitude and knowlwdge was significative with TAFES |
| [42] | Weinstein 2016 | Yes |  | -Dynamic (Need satisfaction Basic psychological need Relieves the frustrations linked to the autonomy competences and relationships needs)  -Control: no treatment | 41  24  17 | Refugees | Siria | Need frustration  PTSD  ditress  Depression | Psychological need scale  PSS  CES-D  STAI  17 items self reported | -Dynamic (Need satisfaction)  7 sessions, 24 minutes, daily | Depression and stress decreased with one week long intervention but not significately and alleviated need frustration. |
|  | Single prospective cohorte trials |  |  |  |  |  |  |  |  |  |  |
| [43] | Arntz 2013 | Yes | 3 mm | -CBT (ImRs: imagination before trauma, if the patient feldt strong enough, rescripting assisted by therapist)  -Control (Partecipans are treated as control theyself) | 22  11/10  11/10 | Refugees |  | Depression  PTSD | SCID1  BDI2  PSS | -CBT (ImRs)  10 sessions, 2-3 exercises | PTSD, significant effects maintained at follow up  Depression: significant effect maintained at follow up |
| [44] | Boehnlein 2004 |  | Each 3 mm | -Combined Pha  (supportive psychotherapy, madication management, antidepressant) | 23 | Refugees | Cambogia | PTSD  Depression  Social, family, work functions  Global function | CAPS  HAM-D  Sheehan Disability Scale  GAF  Quality of life adapted from Visual analogue mood scale | -Combined Pha  10 years | PTSD  Depression  Global function  60% patients improved |
| [45] | Brune 2014 |  |  | -Combined Pha refugees (psychodynamic and CBT and antidepressant, anxiolytic, hipnotic))  -Combined Pha no legal status (psychodinamic and CBT and antidepressant, anxiolytic, hypnotic)) | 190  121  69 | Refugees Asylum seekers | Yugoslavia  America Latina  Turchia  Africa  Iraq  Russia | Depression  Psychosocial distress | HAM-D  CGI | -Combined Pha  daily,  pharmachological treatment, 22 months | Depression: significant results at end therapy for refugees. Psychosocial distress: no significant differences within and between |
| [46] | Carlsson 2010 |  | 9 mm  23 mm | -Combined Psy (psychodinamic, social counselling, physiotherap, medical assistance, some serotonin) | 69/62 | Refugees | Iraq | Depression  Anxiety  Distress  PTSD  Quality life  Event impact | HTQ,  HAM-D HSCL25 WHOQOL  brief |  | Quality life: significant differences at 9 mm  Anxiety: significant differences between 9-23 mm.  Significant differences at 23 mm for all measures in 1/3 patients |
| [47] | D’Ardenne 2007 |  |  | -CBT refugees interpeter trauma focalization, exposition)  -CBT refugees no interpeter (trauma focalization, exposition)  -CBT asylum seekers (trauma focalization, exposition) | 112  36  31  45 | Asylum seekers  Refugees |  | PTSD  Event impact  Depression  Quality life | IES  BDI Manchester short assessment quality life | -CBT (focalization, exposition)  9.1 sessions, 60 minutes, weekly | Depression: significant differences for all groups, more in asylum seekers.  Event impact: significant differences for all groups, more for asylum seekers.  Qality life: significant differences for refugees no interpreter and asylum seekers |
| [48] | Drozdek 1997 |  | 6 mm  36 mm | - Combined Psy (psychotherapy group, dynamic and CBT)  -Combined Pha (group, dynamic and CBT and anxiolytics-tricyclics)  -Pharmachological treatment (anxiolytics and tricyclics)  -Other 3 groups Pahases therapy:  1 Group became psychodynamic, phase oriented, supportive group, focalization and stabilization  2 integrate traumatic memories and affects  3 changes aspects of migration | 60  10  10  10  10 | Refugees | Bosnia | PTSD | Watson questionnaire | -Combined Psy and Pha  48 sessions, beweekly | PTSD: significant positive correlaton in the whole sample between self rated measurements of current psychological wellbeing and PTSD after 36 mm, correlation found also in treated patients |
| [49] | Drozdek 2014 |  | 12 mm  24 mm | -Combined Psy 3*3  -Combined Psy 3*2  -Combined Psy 2*2  2 nonverbal  5 phases:  1 norms, values of group treatment, psychoeducation, alliance, assessment of problems, treatment goals and symptoms.  2 presentations, damage core beliefs, fear of loss control, guilt, shame, grief, acknowledgement, resilience.  3 telling the trauma story, exposure and cognitive restructuring  4 reconnecting the present with past and future, damage core beliefs, roles and identity, coping strategies, current worries and future outlook, resilience.  5 psychoeducation, relapse prevention, treatment evaluation, farewell ritual. (employed dinamic and behavior tecniques) | 69/66  (all) | Refugees Asylum  seekers | Iran Afghanistan | PTSD  Depression  Anxiety | HTQ  HSCL20 | -Combined Psy 85 sessions:  1 phase 10 sessions  2 phase 20 sessions  3 phase 10 sessions  4 phase 30 sessions  5 phase 15 sessions  Specifically:  Group therapy 85 sessions, 90 minutes, daily  No verbal therapy  85 sessions, 75 minutes, daily  -Combined Psy 3*3  No verbal psychotherapy, 3 sessions (psychomotor body therapy, art therapy, music therapy) Group therapy, 2 sessions, 3 days week  -Combined Psy 3*2 same thing of 3*3 but 2 days week)  -Combined Psy 2*2 psychotherapy 2 sessions  Group therapy 1 sessions, 2 days week  -Dynamic 1*1  Support, 1 sessions, weekly | PTSD, Anxiety, Depression significant reduction for 3*3 treatment.  Anxiety significant differences in refugees. |
| [50] | Folkes 2002 |  |  | -CBT (TFT: set rate disease and follow tapping) | 61/31 | Refugees Asylum seekers | Etiopia  Eritrea  Somalia | PTSD  Anxiety  Avoidance  hypervigilance | PCL-C | -CBT (TFT)  30 sessions, 1-3 minutes, daily | Anxiety  Avoidance  Hypervigilance  Significant difference after treatment. |
| [51] | Halvorsen 2010 |  | 1 mm  6 mm | -NET (no verbal exposition all traumetic event, the byography is recorded and corrected) | 16 | Refugees Asylum seekers | Afghanistan  Eritrea  Kosovo  Etiopia  Iran  Sudan  Togo  Iraq | PTSD  Depression | CAPS  HAM-D  Sociodemographic questionnaire | -NET  3sessions | PTSD: significant difference pre-post treatment, pretreatment-follow up and post treatment-follow up  Depression: significant difference between pretreatment- follow up.  Avoidance: significant difference pre-post treatment, pretreatment-follow up.  Hyperarousal: significant difference between pretreatment-follow up |
| [52] | [Naser Morina](https://www.ncbi.nlm.nih.gov/pubmed/?term=Morina N%5BAuthor%5D&cauthor=true&cauthor_uid=22893834) 2012 |  | 3 mm | -Combined Psy (NET and biofeedback) | 18/15 | Refugees | Turchia  Bosnia  Sri Lanka  Iraq  Syria  Vietnam | Psychiatric disorder  Panic  PTSD  Quality life  motivation | MINI  Verbal rating scale  Pain disability index.  CAPS.  WHO self reported EUROHIS-QOL  Visual analogue scale | -Combined Psy  NET 10 sessions  Biofeedback 10 session | Motivation: significant differences post biofeedback |
| [53] | Palic 2009 |  | 6 mm | -Combined Pha (CBT, EMDR, CTR: stabilization, coming to terms with trauma, integration of traumatic memories and grieving, body awareness therapy, education on body awareness in coping with pain and stress, SSRI and or antipsychotic) | 26 | Refugees |  | PTSD  Global functioning  Psychological distress  Somatization  Physic parameters  Development postraumatic symptoms  Cognitive changing and brain damage | HTQ  GAF  TSC 33  SCAN  CSS  TMT | -Combined Pha  16 sessions | PTSD (HTQ TSC) significant decreased after treatment and follow up  Avoidance and numbing (HTQ): significant decreased post treatment and follow up  Negative affectivity (TSC): significant decreased after treatment and follow up  Somatization (TSC): significant decreased after treatment  Support (CSS): significant increased post treatment and follow up  Global functioning (GAF) significant increased after treatment and follow up |
| [54] | Pocock 2018 |  |  | -Dynamic (post traffiking service) | 275 (quantitative sample)  198/77  (qualitative sample) |  | Birmania  Thailandia  Laos  Vietnam | Depression  Anxiety  Physical symptoms  Occupational hazard exposures and presences of personal protective equipment for that hazard. | Miller abuse physical symptoms and injury scale for abuse specific health problems  Occupational health risks  HSCL 25 |  |  |
| [55] | Raghavan 2013 |  | 6 mm  18 mm | -Combined Pha (medicine program of survivors of torture: individual and group therap, counselling, free medicine) | 178/172 | Refugees Asylum seekers | Europa  America  Africa | General symptoms  PTSD  Depression  Anxiety  Somatizzation | HTQ  BSI | -Combined Pha  7.5 sessions, daily | Depression  Anxiety  Somatizatiom  PTSD  significant differences at 6 mm |
| [56] | Rees 2014 |  | 10 dd  1 mm  (assessment baseline: 0-1-3 mm) | -Dynamic  (TM, mantra) | 11 | Refugees | Congo  Kampala  Uganda | PTSD | PCL-C | -Dynamic (TM) instruction 10 sessions, 20 minutes, daily | PTSD PCL scores significantly decreasesing frome the last baseline to 30 day YM assessment |
| [57] | Schulz 2006 |  |  | -CBT (CPT) cognitive therapy, written exposure | 53/44 | Refugees | Bosnia  Afghanistan | PTSD | PSS | -CBT (CPT)  17 sessions which included three to four assessment sessions before CPT, 120 minutes | PTSD significant decrease |
| [58] | Sierra Van Wyk  2012 |  | 6,9 mm | -Combined Psy (psychoeducation, structured skills based therapy, expressive therapy, supportive therapy, couples and family therapy, CBT, exposition) | 70/62 | Refugees | Burma | PTSD  Depression  Anxiety  Somatizzation | HTQ  HSCL 37  Post Migration Living  Difficulties Checklist | -Combined Psy  2-3 sessions, 120-150 minutes | PTSD  Depression  Anxiety  Somatization:  Significant decrease symptoms |
| [59] | Stammel 2017 |  | 6,6 mm | -Combined Psy (multidisciplinary treatment traumatized refugees naturalistic setting:  Phase 1, Diagnosis;  Phase 2, stabilization;  Phase 3, trauma focused;  Phase 4, future oriented;  Phase 5, self evaluation | 76/39 | Refugees  Asylum seekers | Iran  Ira  Kurdosh  Syria  Kosovo  Afghanistan  Armenia  Kenya  Angola  Chile  Lebanon | PTSD  Depression  Anxiety  Somatization  Psychiatric disorders  Quality life | PDS  HSCL 25  SCL 90  EUROHIS QOL 8  MINI | -Combined Psy  30 sessions | PTSD  Depression  Anxiety  No significant improvement  Somatization:  significant improvement  Quality life:  significant improvement over time |
| [60] | Tay 2019 |  |  | -Combined Psy Integrative Adapt Therapy (psychoeducation, trauma narrative exposure, problem solving, stress management, emotion regulation, cognitive reappraisal, meaning making, behavioural activation (ptional), strengthen social support (optional) | 70 | Refugees | Rohingya  (Asia) | PTSD  Depression  Anxiety  Adaptation and development after persecution and trauma model: Impact events  Traumatic loss  Injustice sense.  Complicated grief  Explosive anger  Comorbidity mental disorder. | Refugees mental health assessment package  Adaptive stress index  Adapt meter | -Combined Psy  45 minutes | PTSD  Depression  Anxiety  Impact event  Traumatic loss  Injustice sense  Complicated grief  Explosive anger  Comorbidity  improve capacity to adapt to serial psychosocial changes in refugee life. |
| [61] | Tran 2014 |  |  | -Combined Psy (ALMA: mental health, stress and coping skills and group wiyh promotoras) | 58/32 |  | South  America | Depression  Stress perceive  Social support | CES-D  PARC-D  PSS  SMPSS | -Combined Psy  6 sessions, 120-150 minutes, weekly and group 4-9 sessions, once month | Depression  Stress:  significant decrease  Social support:  significant increases |
| [62] | Weine 1999 |  | 6 mm | -Dynamic (testimony psychotherapy: multiethnic, family, life experienced history) | 20 | Refugees | Bosnia | PTSD  Depression  Global functioning  Avoidance  hyperarousal  General symptomatology | PSS  BDI  GAF | -Dynamic  6 sessions, 90 minutes, 30-96 pages in 5 units of narration, weekly or beweekley | PTSD  Depression  Avoidance  Hyperarousal:  Significant decrease  Global functioning:  Significant increases |
| [63] | Whitsett 2017 |  | 12 mm | -Combined Psy  (group and individual therapy: CBT, psychoeducation, combing suppoortive, interpersonal and exposure techniques, sleep hygiene, relaxation, cognitive restructuring, social support) | 105 | Asylum seekers | Azerbaijan Burkina Faso Burundi Cameroon Central African Republic of Congo Eritrea Ethiopia Liberia Mali Nepal Pakistan Russia Rwanda Sierra Leone Togo Uganda | PTSD  Anxiety  Depression  Torture | HURIDOCS  HTQ  HSCL 25 | -Combined Psy psychotherapy and group therapy, Supportive, psychoeducation, CBT and esposition and reprocessing trauma, weekly | PTSD  Depression  Anxiety:  Significant reduction |
| [64] | Zehetmair 2018 |  | 1 mm | -CBT (immaginative stabilization group, body scan) | 46-43 | Refugees |  | PTSD  Depression  Anxiety  Anhedonia  Distress  Arousal  Dominance  Valence | Primary care PTSD screen for DSM5  Patient health questionnaire  General anxiety disorder questionnaire  Self assessment manikin scale  Distress thermometer of refugee health screener 15 | -CBT  86 sessions | Anxiety: Significant improvement  Distress: significant reduction  Dominance and valence: significant improvement |

RCT designe: age: 19-51 yy

Multiple cohort trial designe: age: 18-70 yy

Single cohort trial designe: age 18-80

All studies to consider male and female population

ADIS: Anxiety disorders interview schedule; ALMA: assessment and intervention latin friens, network of women and 3 are trained propmoters; ANPs: assessment neuropsychiatric; AQ: avoidance questionnaire; ASI: addiction severity index; AUDIT: Alcohol use disorder identification test; BDI: Beck depression inventory; Beck SSI: scale for suicidal ideation; BSI: brief symptom inventory; CAFES: Coffee and family education and support; CAPS: clinician administered PTSD scale; CASP: contact and safety planning; CBT: cognitive behavior treatment; CES-D: Center for Epidemiologic Studies Depression Scale; CESD-R: Center for Epidemiologic Studies Depression Scale revised; CFESDS: centre for epydemiologichal studies depression scale; CGI: clinical global impression; CIDI: composit international diagnostic interview; Combined Pha: combined psychological with pharmacological treatments; Combined Psy: combined psychological treatments; CPT: cognitive processing therapy; CR: cognitive restructuring; CROP: group facilitator for culture sensitive and resources oriented peer; CSI: clinician structured interview; CSS: chrisis support scale; CTS: conflict tactis scale; CVDTE: checklist of war detention and torture events; DFMQ: demography of forced migration questionnaire; EMDR: eye movement desensitization and reprocessing; EMDR-R-TEP: eye movement desensitization and reprocessing recent traumatic episode protocol; ERS: environment rating scale; EUROHIS-QOL GAF: global assessment of functioning; GFI: global severity index FHI: Family Hardiness Index; FPSC: Family Problem Solving Communication Index; GAS: goal attainment scale; h: hours HAM-A: Hamilton Anxiety rating scale; HAM-D: Hamilton depression rating scale; HRSD: Hamilton rating scale depression; HSCL (20,25): Hopkins Symptom Checklist; HTQ: Harvard Trauma Questionnaire; HURIDOCS: events standard formats : a tool for documenting human rights violations; IES: impact of event scale; IES-R: impact of events scale revised; IPT: interpersonal psychotherapy; ISEL: Interpersonal Support Evaluation List; MEG-ssVEF: magnetoencephalography steady-state visual evoked fields; MG: monitoring group; MINI: mini international neuropsychiatric interview; NET: narratives experiences treatment; N-FSS: neural flashbacks severity scale; N-PASS: neural parameters severity scale; OFSS: orthostatic functions severity scale; OPAFSS: orthostatic parameters flashbacks severity scale; OPAI: orthostatic parameters interview; OPASS: orthostatic parameters severity scale; PARC-D: patients attitude toward and ratings of care for depression scale; PCL: ptsd checklist; PCL-C: ptsd checklist civilian version; PDS: posttraumatic stress diagnostic scale; PHQ-15: patient health questionnaire; PS: physical symptomatology; PSOT: program survivors of torture; PSS: physical symptom score; PSS-SR: physical symptom score self report; PTGI: posttraumatic growth inventory; PTSD: posttraumatic stress disorder; PTSDSS: ptsd symptom scale; PTSS: posttraumatic stress syndrome; QL: quality life; QOLI: quality of life inventory; RI: reactivity index; ss: subjects; SASB: Sustainability Accounting Standards Board; SCAN: clinical assessment in neuropsychiatry; SCID-5: structured clinical interview for DSM.5; SCL-90: symptom checklist; SDS: Sheehan Disability Scale; SF: social functioning; SF-MPQ: short-form McGill Pain Questionnaire; SF12: self report form12; SFS: skills functions scale; SIT: stress inoculation training ; SM: stress management; SMSSP: multidimensional scale perceive social support; SPRINT: Short PTSD Rating Interview; SRQ20: self reporting questionnaire; SSI (in WHO): supplemental security income; STAI: State-Trait Anxiety Inventory; STAXI: State-Trait Anger Expression Inventory; SUPREME MISS: multisistemic intervention for suicide behaviour; TAFES: Tea and Families Education and Support; TAU: treatment as usual; TC: trauma counselling; TEI: traumatic event impact; TF-CBT: trauma focused cognitive behavioural treatment; TFT: Thought Field Therapy; TFT: trauma focalized treatment; TM: trascendental meditation; TMT: trail making test; TSC: Trauma symptom checklist; VAS: visual analogue scale; VCOV: Checklist organized violence; WB: well being; WB-FACES-PRS 5: wong baker faces pain rating scale; WHO5: world health organization scale assessment; WHODAS2.0: WHO Disability Assessment Schedule 2.0; WHOQOL(5): WHO quality of life; WLC: waiting list control;

Table 2s. Results of the qualitative analysis applied to systematic review that summarized the ratio significant vs. not significant efficacious trials considering the kind of the treatment and the outcome. The trials included in the analysis were 89/107

| Treatments | Studies: 52  Samples 107: 89 included in results, 18 excluded from results | Depression  (trials 61) | | Anxiety  (trials 36) | | Somatization  (trials 24) | |
| --- | --- | --- | --- | --- | --- | --- | --- |
|  |  | Significant | Not significant | Significant | Not significant | Significant | Not significant |
| CBT | 22 samples 28 trials | 11 | 7 | 4 | 4 | 0 | 2 |
| NET | 9 samples 9 trials | 4 | 3 | 0 | 0 | 1 | 1 |
| Dynamic | 15 samples 16 trials | 5 | 5 | 2 | 2 | 1 | 1 |
| Combined.Psy | 20 samples 31 trials | 6 | 7 | 6 | 7 | 3 | 2 |
| Combined Pha | 23 samples 37 trials | 8 | 5 | 4 | 7 | 5 | 8 |
| Usual treatment/waiting list | 16 samples and 16 trials (excluded) | / | / | / | / | / | / |
| Pharmacological treatments | 2 samples and 2 trials (excluded) | / | / | / | / | / | / |
| Total traials | 107 samples and 134 trials | 34 | 27 | 16 | 20 | 10 | 14 |


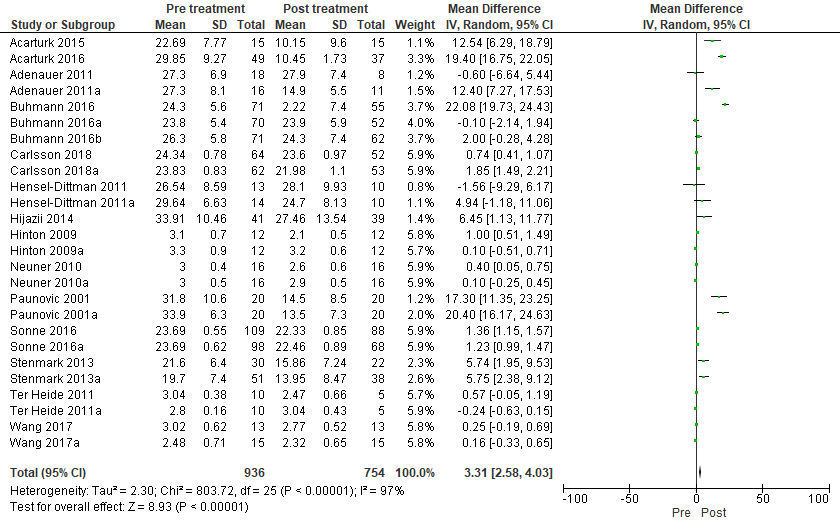

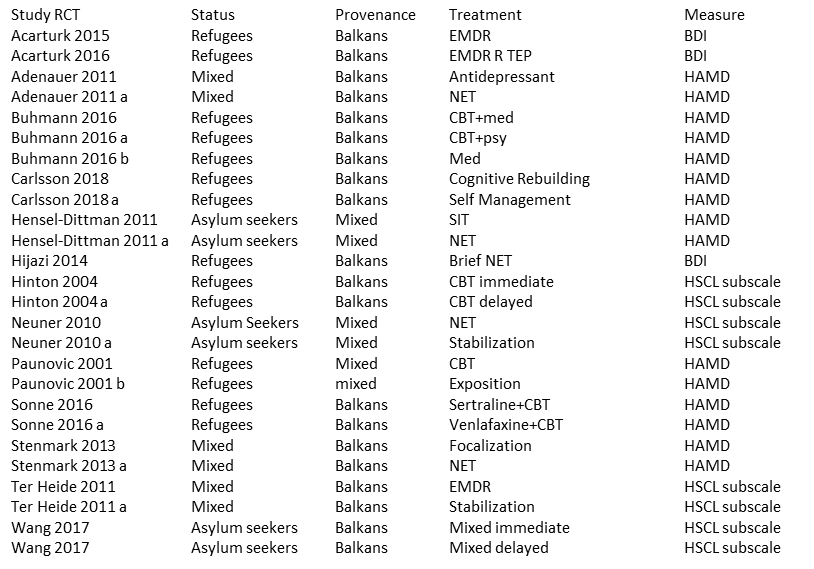


**Submetanalysis results of the despression outcome divided for status provenance and kind of treatment**

*Submetanalysis results on the kind of status for depression RCT*:

Refugees

714-585 ss – 8 studies – 14 trial. Tau^2^ 3.28; Chi^2^ 640.06 df=13 p<0.00001; I^2^ 98%; Z 9.34 p<0.00001

Asylum seekers

87-80 ss – 3 studies – 6 trial. Tau^2^ 0.00; Chi^2^ 3.99 df=5 p<0.55; I^2^ 0%; Z 2.36 p<0.02

*Submetanalysis results on the kind of provenance for depression RCT*

Middle East

837-662 ss – 11 studies – 20 trial. Tau^2^ 2.34; Chi^2^ 654.76 df=19 p<0.00001; I^2^ 97%; Z 7.75 p<0.00001

*Submetanalysis results on the kind of treatment for depression RCT*

CBT

213-162 ss – 7 studies – 8 trial. Tau^2^ 5.15; Chi^2^ 230.88, df=7, p<0.00001; I^2^ 97%; Z 4.02 p=0.0001

NET

138-114 ss – 5 studies – 5 trial. Tau^2^ 23.22; Chi^2^ 37.02 df=4 p<0.00001; I^2^ 89%; Z 2.4 p=0.02

Combined psychological with pharmacological treatments

539-442 ss – 5 studies – 10 trial. Tau^2^ 2.30; Chi^2^ 444.14 df=9 p<0.00001; I^2^ 98%; Z 7.44 p<0.00001

Figure 1s. Forest plot with table of the RCT studies for depression: 14 studies, 26 trial, 936 participants pre treatment and 754 s post treatment


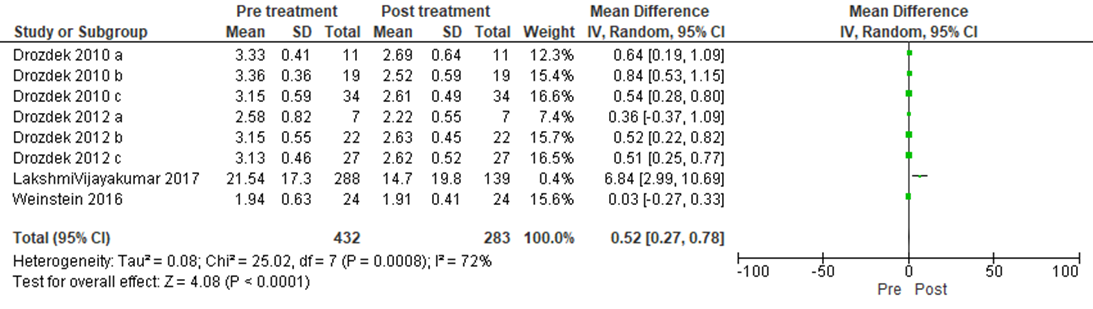


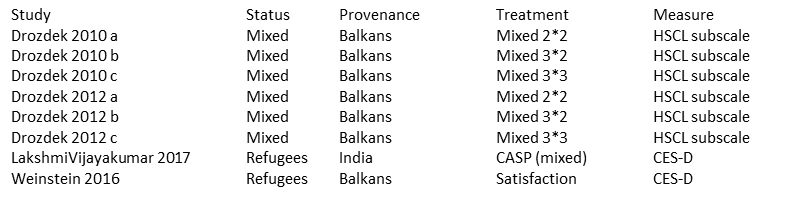


Figure 2s: forest plot and table of the multiple perspective cohort trial studies on depression. 4 studies, 8 trial and 432 participants pre treatment and 283 participants post treatment.


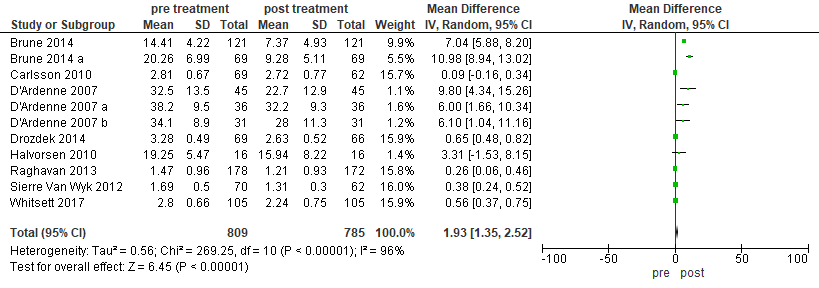


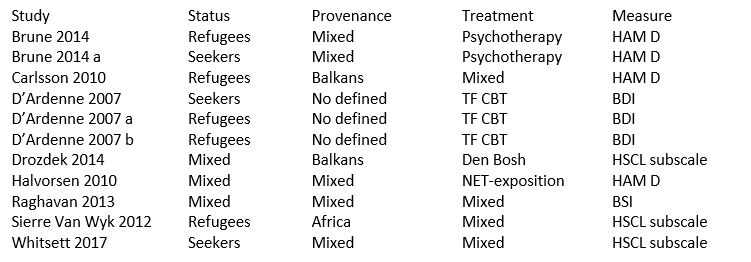


**Submetanalysis results of the depression SCT divided for status and kind of treatments**

*Submetanalysis results for kind of status for depression SCT*

Refugees

327-312 ss – 4 studies – 5 trial. Tau^2^ 1,40; Chi^2^ 143,98 df=4 p<0.00001; I^2^ 97%; Z 4,03 p<0.0001

*Submetanalysis results for kind of treatment for depression SCT*

Combined psychological treatment

313-295 ss – 4 studies - 4 trial. Tau^2^ 0.04; Chi^2^ 15.59 df=3 p=0.001; I^2^ 81%; Z 4.08 p<0.0001

Figure 3s: forest plot and table of the single perspective cohort trial studies on depression. 8 studies, 11 trial, 809 participants pre treatment and 785 participants post treatment.


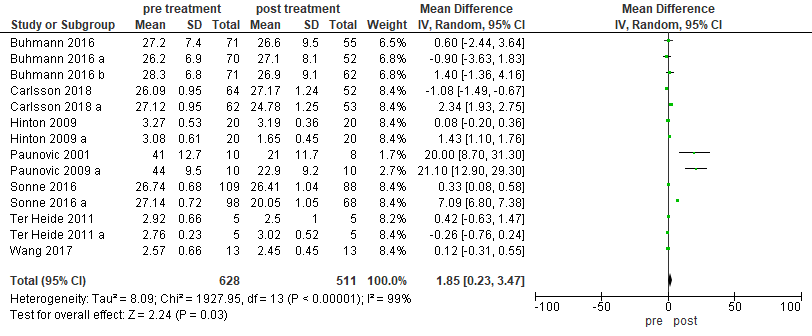


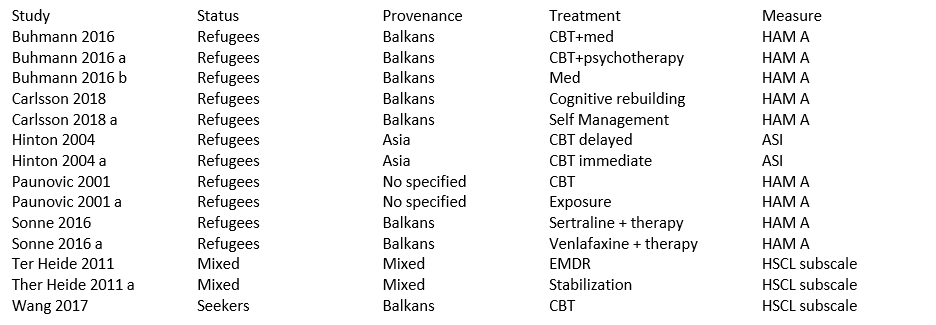


Figure 5: forest plot and table to the RCT studies on Anxiety. 7 studies, 14 trial, 628 ss pre treatment and 511 ss post treatment. Excluded 2 trial paunovic because BDI applyed to same sample. we mantained HAMD for omogeneity

**Submetanalysis divided for status, provenance and kind of treatment**

*Submetanalysis for kind of status on anxiety RCT*

Refugees

605-488 ss – 5 studies – 11 trial. Tau^2^ 9.31; Chi^2^ 1813.09 df=10 p<0.00001; I^2^ 99%; Z 2.49 p=0.01

*Submetanalysis for kind of provenance on anxiety RCT*

Middle East

558-443 ss – 4 studies – 8 trial. Tau^2^ 12.49; Chi^2^ 1664.77 df=7 p<0.00001; I^2^ 100%; Z 1 p=0.32

*Submetanalysis for kind of treatment on anxiety RCT*

Combined psychological with pharmacological treatments

535-436 - 5 studies – 10 trial. Tau^2^ 9.32; Chi^2^ 1809.25 df=9 p<0.0001; I^2^ 100%; Z 2.69 p=0.007

Figure 4s. Forest plot with table of the RCT studies for anxiety. 7 studies, 14 trial, 628 participants pre treatment and 511 participants post treatment.


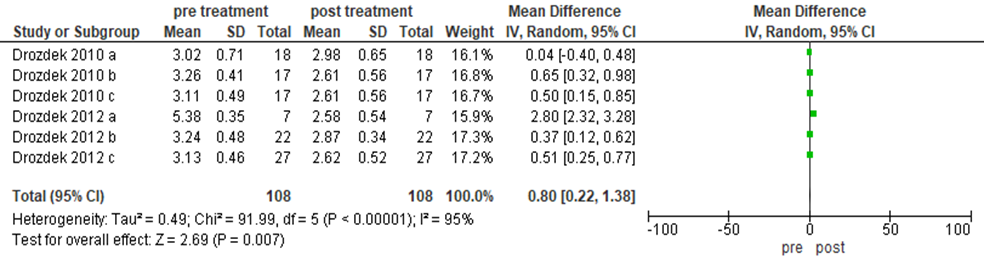


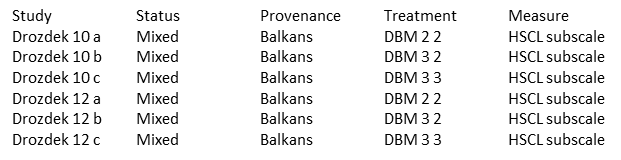


Figure 5s. forest plot for multiple perspective cohort trial on anxiety. 2 works, 6 trial,108 participants pre and post treatment


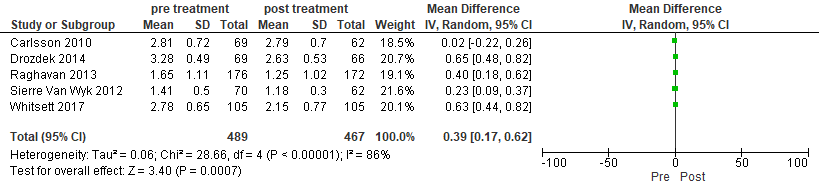


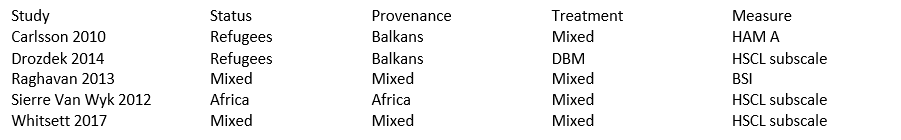


Figure 6s: forest plot for single perspective cohort trial studies on Anxiety. 5 studies, 5 trial, 489 participants pre treatment and 467 participants post treatment


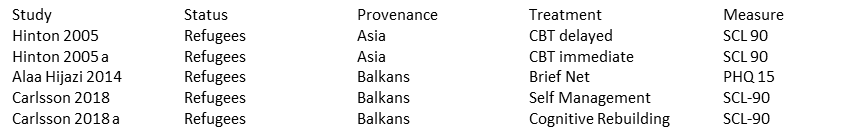

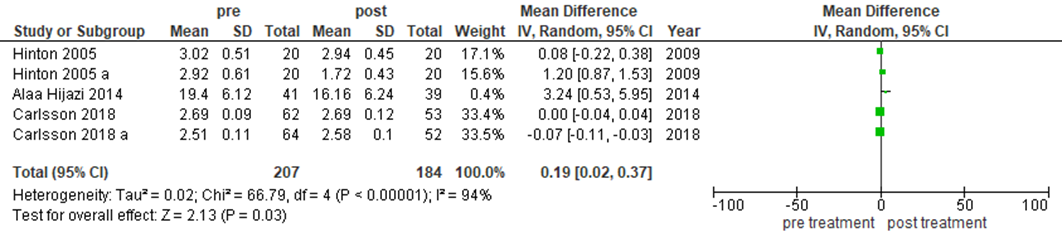


Figura 7s. Forest plot for RCT studies on somatization. 3 studies, 5 trial, 207 participants pre treatment and 184 participants post treatment

Funnel Plots
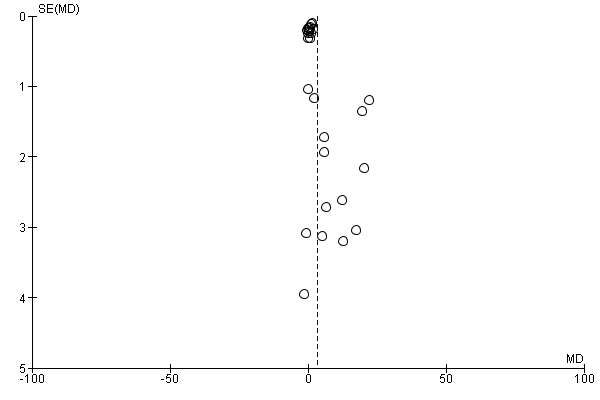


Figure 8s: Funnel plot that represent the bias of the metanalysis applied to the depression outcome. RCT design


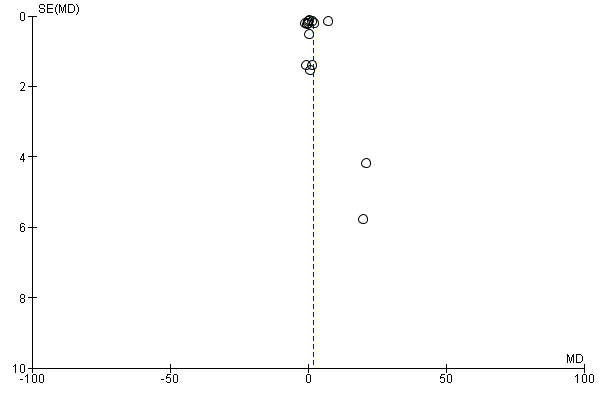


Figure 9s: Funnel plot that represent the bias of the metanalisys applied to the anxiety outcome. RCT design

Table 3s: Cochraine risk of bias tool scale applied to systematic review for studies with RCT design, the studies cued in “other sources of bias” section were excluded from metanalysis caused of data unclear.

|  | Random sequence generation | Allocation concealment | Selective reporting | Blinding partecipants and personnel | Blinding outcome assessment | Other sources of bias |
| --- | --- | --- | --- | --- | --- | --- |
| Acaturk et.al. 2015 | + | + | + | - | + |  |
| Akarturk et.al. 2016 | + | + | + | - | + |  |
| Adenauer et.al 2011 | + | + | + | + | + |  |
| Buhmann et.al. 2016 | + | + | + | - | + |  |
| Carlsson et.al. 2018 | + | + | + | + | + |  |
| Hensel-Dittmann et.al. 2011 | + | + | + | + | ? |  |
| Hijazi Alaa et.al. 2014 | + | + | + | ? | ? | * |
| Hinton et.al 2004 | + | ? | + | ? | ? |  |
| Hinton et.al. 2005 | + | ? | + | ? | ? |  |
| Hinton et.al. 2009 | ? | ? | + | ? | ? | * |
| Meffert et.al. 2014 | + | + | + | - | ? | * |
| Neuner at.al 2004 | + | + | + | + | + | * |
| Neuner et.al. 2008 | + | ? | + | + | ? | * |
| Neuner et.al. 2010 | + | + | + | + | + |  |
| Nordbrant et.al. 2015 | + | - | + | + | + | * |
| Paunovic and Lars Goran 2001 | + | ? | + | ? | ? |  |
| Renner et.al. 2011 | + | ? | + | ? | ? | * |
| Sonne et.al. 2016 | + | + | + | - | + |  |
| Stenmark et.al. 2013 | + | ? | + | + | ? |  |
| Ter Heide et.al. 2011 | + | + | + | ? | ? |  |
| Wang et.al. 2017 | + | + | + | + | + |  |
| Weine et.al. 2008 | + | ? | - | ? | ? |  |

Legend: + (low risk); - (high risk); ? (unclear)

Table 4s: Newcastle Ottawa Quality assessment scale cohort studies scale applied to studies with MCT and SCT design included in systematic review and in metanalysis.

| *Multiple perspective cohort trial* | Representativeness of exposed cohort | Selection of the non exposed cohort | Ascertainment of exposure | Demonstration that outcome was not presenta at start of study | Comparability of cohorts on the basis of the design or analysis | Assessment of outcome | Was follow up long enough for outcomes to occur | Adequacy of follow up cohorts |
| --- | --- | --- | --- | --- | --- | --- | --- | --- |
| Drozdek et.al 2010 | + | + | ++ | ++ | ++ | + | ++ | ++ |
| Drozdek et al 2012 | + | + | ++ | ++ | ++ | + | ++ | ++ |
| Kruse et al 2009 | + | + | ++ | ++ | ++ | + | ++ | + |
| Lakshmi Vijaayakumar et al 2017 | + | + | + | ++ | + | + | ++ | + |
| Rees 2013 | + | + | ++ | ++ | + | + | + | + |
| Schaal et.al. 2009 | - | + | ++ | ++ | - | ++ | ++ | ++ |
| Weine et.al. 2003 | + | ++ | - | ++ | ++ | + | + |  |
| Weinstein et.al.2016 | + | ++ | + | ++ | ++ | + | + |  |
| -*Single perspective cohort trial* |  |  |  |  |  |  |  |  |
| Arntz et.al. 2013 | + | + | ++ | ++ | - | ++ | + | ++ |
| Boehnlein et.al 2004 | + | + | ++ | + |  | ++ | + | ++ |
| Brune et.al. 2014 | + | + | - | + + |  | ++ |  |  |
| Carlsson et.al. 2010 | + | + | - | ++ |  | ++ | ++ | + |
| D’Aerdenne et.al. 2007 | + | + | + | ++ |  | + |  |  |
| Drozdek et.al. 1997 | + | + | ++ | ++ |  | + | ++ | + |
| Drozdek et.al. 2014 | + | + | + | ++ |  | ++ | ++ |  |
| Folkes et.al. 2002 | + | + | - | ++ |  | + |  |  |
| Halvorsen et al. 2010 | + | + | + | ++ |  | ++ | ++ | ++ |
| Morina et.al.2012 | + | + | ++ | ++ |  | ++ | + |  |
| Palic et.al. 2009 | + | + | ++ | ++ |  | ++ | ++ | ++ |
| Pocock et.al.2018 | + | + | ++ | + |  | + |  |  |
| Raghavan et al. 2013 | + | + | ++ | ++ |  | + | ++ | ++ |
| Rees et.al. 2014 | + | + | + | ++ |  | + | + | ++ |
| Schultz et.al. 2006 | + | ++ | ++ | + |  | ++ |  |  |
| Sierre Van Wyk et.al. 2012 | + | + | ++ | ++ |  | + | ++ | + |
| Stammel et.al.2017 | + | + | ++ | ++ |  | ++ | ++ | + |
| Tay et.al.2019 | + | ++ | ++ | + |  | - |  |  |
| Tran et.al. 2014 | - | ++ | ++ | ++ |  | ++ |  |  |
| Weine et.al 1999 | + | + | ++ | ++ |  | + | ++ |  |
| Whitsett et al. 2017 | + | + | ++ | ++ |  | - | ++ |  |
| Zehetmair et.al. 2018 | + | + | ++ | ++ |  | ++ | ++ | + |

Legend: ++ (low bias risk; + moderate bias risk; -high bias risk; white square (data unclear or absent)

**References**

1. Lely JC, Smid GE, Jongedijk RA, W Knipscheer J, Kleber RJ. The effectiveness of narrative exposure therapy: a review, meta-analysis and meta-regression analysis. European journal of psychotraumatology. 2019; 10: 1550344.
2. Williams ME, Thompson SC. The use of community-based interventions in reducing morbidity from the psychological impact of conflict-related trauma among refugee populations: a systematic review of the literature. Journal of immigrant and minority health. 2011; 13: 780-794.
3. Robjant K, Fazel M. The emerging evidence for narrative exposure therapy: A review. Clin Psychol Rev. 2010; 30: 1030-1039.
4. Eurostat E: gas retail tables H1. European Comission; 2016.
5. Tribe RH, Sendt KV, Tracy DK. A systematic review of psychosocial interventions for adult refugees and asylum seekers. Journal of Mental Health. 2019; 28: 662-676.
6. UNHCR W UNAIDS. Policy statement on HIV testing and counselling in health facilities for refugees, internally displaced persons and other persons of concern to UNHCR. Geneva; 2014.
7. UNHCR R. Syria regional refugee response. UNHCR Inter-agency Information Sharing Portal. Turkey; 2016.
8. Gwozdziewycz N, Mehl-Madrona L. Meta-analysis of the use of narrative exposure therapy for the effects of trauma among refugee populations. The Permanente Journal. 2013; 17: 70.
9. Mahoney A, Karatzias T, Hutton P. A systematic review and meta-analysis of group treatments for adults with symptoms associated with complex post-traumatic stress disorder. Journal of affective disorders. 2019; 243: 305-321.

10. Pompili M, Gibiino S, Innamorati M, Serafini G, Del Casale A, De Risio L, Sher L. Prolactin and thyroid hormone levels are associated with suicide attempts in psychiatric patients. Psychiatry research. 2012; *200*(2-3): 389-394.

11. Pompili M, Shrivastava A, Serafini G, Innamorati M, Milelli M, Erbuto D, Lester D. Bereavement after the suicide of a significant other. Indian Journal of Psychiatry. 2013; *55*(3): 256.

1. Slobodin O, de Jong JT. Family interventions in traumatized immigrants and refugees: a systematic review. Transcultural Psychiatry. 2015; 52: 723-742.
2. Acarturk C, Konuk E, Cetinkaya M, Senay I, Sijbrandij M, Cuijpers P, Aker T. EMDR for Syrian refugees with posttraumatic stress disorder symptoms: Results of a pilot randomized controlled trial. European Journal of Psychotraumatology. 2015; 6: 27414.
3. Acarturk C, Konuk E, Cetinkaya M, Senay I, Sijbrandij M, Gulen B, Cuijpers P. The efficacy of eye movement desensitization and reprocessing for post-traumatic stress disorder and depression among Syrian refugees: Results of a randomized controlled trial. Psychol Med. 2016; 46: 2583-2593.
4. Adenauer H, Catani C, Gola H, Keil J, Ruf M, Schauer M, Neuner F. Narrative exposure therapy for PTSD increases top-down processing of aversive stimuli-evidence from a randomized controlled treatment trial. BMC neuroscience. 2011; 12: 127.
5. Buhmann CB, Nordentoft M, Ekstroem M, Carlsson J, Mortensen EL. The effect of flexible cognitive–behavioural therapy and medical treatment, including antidepressants on post-traumatic stress disorder and depression in traumatized refugees: pragmatic randomized controlled clinical trial. The British Journal of Psychiatry. 2016; 208: 252-259.
6. Carlsson J, Sonne C, Vindbjerg E, Mortensen EL. Stress management versus cognitive restructuring in trauma-affected refugees—A pragmatic randomized study. Psychiatry Rec 2018; 266: 116-123.
7. Hensel-Dittmann D, Schauer M, Ruf M, Catani C, Odenwald M, Elbert T, Neuner F. Treatment of traumatized victims of war and torture: a randomized controlled comparison of narrative exposure therapy and stress inoculation training. Psychother Psychosom. 2011; 80: 345-352.
8. Hijazi AM, Lumley MA, Ziadni MS, Haddad L, Rapport LJ, Arnetz BB. Brief narrative exposure therapy for posttraumatic stress in Iraqi refugees: A preliminary randomized clinical trial. J Trauma Stress. 2014; 27: 314-322.
9. Hinton DE, Pham T, Tran M, Safren SA, Otto MW, Pollack M H. CBT for Vietnamese refugees with treatment‐resistant PTSD and panic attacks: A pilot study. J Trauma Stress: Official Publication of The International Society for Traumatic Stress Studies. 2004; 17: 429-433.
10. Hinton DE, Chhean D, Pich V, Safren SA, Hofmann SG, Pollack MH. A randomized controlled trial of cognitive‐behavior therapy for Cambodian refugees with treatment‐resistant PTSD and panic attacks: A cross‐over design. J Trauma Stress: Official Publication of The International Society for Traumatic Stress Studies. 2005; 18: 617-629.
11. Hinton DE, Rivera EI, Hofmann SG, Barlow DH, Otto MW. Adapting CBT for traumatized refugees and ethnic minority patients: Examples from culturally adapted CBT (CA-CBT). Transcultural psychiatry. 2009; 49: 340-365.
12. Meffert SM, Abdo AO, Alla OAA, Elmakki YOM, Omer AA, Yousif S, Marmar CR. A pilot randomized controlled trial of interpersonal psychotherapy for Sudanese refugees in Cairo, Egypt. Psychological Trauma: Theory, Research, Practice, and Policy. 2014; 6: 240.
13. Neuner F, Schauer M, Klaschik C, Karunakara U, Elbert T. A comparison of narrative exposure therapy, supportive counseling, and psychoeducation for treating posttraumatic stress disorder in an african refugee settlement. J Consult Clin Psychol. 2004; 72: 579.
14. Neuner F, Onyut PL, Ertl V, Odenwald M, Schauer E, Elbert T. Treatment of posttraumatic stress disorder by trained lay counselors in an African refugee settlement: a randomized controlled trial. J Consult Clin Psychol. 2008; 76: 686.
15. Neuner F, Kurreck S, Ruf M, Odenwald M, Elbert T, Schauer M. Can asylum-seekers with posttraumatic stress disorder be successfully treated? A randomized controlled pilot study. Cognitive behaviour therapy. 2010; 39: 81-91.
16. Nordbrandt M S, Carlsson J, Lindberg LG, Sandahl H, Mortensen EL. Treatment of traumatised refugees with basic body awareness therapy versus mixed physical activity as add-on treatment: Study protocol of a randomised controlled trial. Trials. 2015; 16: 477.
17. Paunovic N, Öst LG. Cognitive-behavior therapy vs exposure therapy in the treatment of PTSD in refugees. Behav Res Ther. 2001; 39: 1183-1197.
18. Renner W, Berry JW. The ineffectiveness of group interventions for female Turkish migrants with recurrent depression. Social Behavior and Personality: an international journal. 2011; 39: 1217-1234.
19. Sonne C, Carlsson J, Bech P, Vindbjerg E, Mortensen EL, Elklit A. Psychosocial predictors of treatment outcome for trauma-affected refugees. European journal of psychotraumatology. 2016; 7: 30907.
20. Stenmark H, Catani C, Neuner F, Elbert T, Holen A. Treating PTSD in refugees and asylum seekers within the general health care system. A randomized controlled multicenter study. Behav Res Ther. 2013; 51: 641-647.
21. ter Heide FJJ, Mooren T, Kleijn W, de Jongh A, Kleber R. EMDR versus stabilisation in traumatised asylum seekers and refugees: Results of a pilot study. European journal of psychotraumatology. 2011; 2: 5881.
22. Wang SJ, Bytyçi A, Izeti S, Kallaba M, Rushiti F, Montgomery E, Modvig J. A novel bio-psycho-social approach for rehabilitation of traumatized victims of torture and war in the post-conflict context: a pilot randomized controlled trial in Kosovo. Conflict and health. 2017; 10: 34.
23. Weine S. Family roles in refugee youth resettlement from a prevention perspective. Child Adolesc Psychiatr Clin N Am. 2008; 17: 515-532.
24. Drožđek B, Bolwerk N. Evaluation of group therapy with traumatized asylum seekers and refugees—The Den Bosch Model. Traumatology. 2010; 16: 117-127.
25. Droždek B, Kamperman AM, Bolwerk N, Tol WA, Kleber RJ. Group therapy with male asylum seekers and refugees with posttraumatic stress disorder: A controlled comparison cohort study of three day-treatment programs. J Nerv Ment Dis. 2012; 200(9): 758-765.
26. Kruse J, Joksimovic L, Cavka M, Wöller W, Schmitz N. Effects of trauma‐focused psychotherapy upon war refugees. J Trauma Stress: Official Publication of The International Society for Traumatic Stress Studies. 2009; 22: 585-592.

38. Lakshimi Vijayakumar L. Challenges and opportunities in suicide prevention in South-East Asia. WHO South-East Asia journal of public health. 2017; 63(7):589-597.

39. Rees S, Silove DM, Tay K, Kareth M. Human rights trauma and the mental health of West Papuan refugees resettled in Australia. Med J Aust. 2013; 199: 280-283.

40. Schaal S, Elbert T, Neuner F. Narrative exposure therapy versus interpersonal psychotherapy. Psychother Psychosom. 2009; 78: 298-306.

41. Weine SM, Raina D, Zhubi M, Delesi M, Huseni D, Feetham S, Pavkovic I. The TAFES multi-family group intervention for Kosovar refugees: A feasibility study. J Nerv Ment Dis. 2003; 191: 100-107.

42. Weinstein N, Khabbaz F, Legate N. Enhancing need satisfaction to reduce psychological distress in Syrian refugees. J Consult Clin Psychol.2016; 84: 645.

43. Arntz A, Sofi D, van Breukelen G. Imagery Rescripting as treatment for complicated PTSD in refugees: A multiple baseline case series study. Behav Res Ther. 2013; 51: 274-283.

44. Boehnlein JK, Kinzie JD, Sekiya U, Riley C, Pou K, Rosborough B. A ten-year treatment outcome study of traumatized Cambodian refugees. J Nerv Ment DisThe. 2004; 192: 658-663.

45. Brune M, José Eiroá-Orosa F, Fischer-Ortman J, Haasen C. Effectiveness of psychotherapy for traumatized refugees without a secure residency status. International Journal of Migration, Health and Social Care. 2014; 10: 52-59.

46. Carlsson JM, Olsen DR, Kastrup M, Mortensen, EL. Late mental health changes in tortured refugees in multidisciplinary treatment. J Nerv Ment Dis. 2010; 198: 824-828.

47. d'Ardenne P, Ruaro L, Cestari L, Fakhoury W, Priebe S. Does interpreter-mediated CBT with traumatized refugee people work? A comparison of patient outcomes in East London. Behavioural and Cognitive Psychotherapy. 2007; 35: 293-301.

48. Drozdek B. Follow-up study of concentration camp survivors from Bosnia-Herzegovina: three years later. J Nerv Ment Dis. 1997; 185: 690-694.

49. Drožđek B, Kamperman AM, Tol WA, Knipscheer JW, Kleber RJ. Seven‐year follow‐up study of symptoms in asylum seekers and refugees with PTSD treated with trauma‐focused groups. J Clin Psychol. 2014; 70: 376-387.

50. Folkes CE. Thought Field Therapy and trauma recovery. Mental health. 2002; 4.

51. Halvorsen JØ, Stenmark H. Narrative exposure therapy for posttraumatic stress disorder in tortured refugees: A preliminary uncontrolled trial. Scand J Psychol. 2010; 51: 495-502.

52. Morina N, Maier T, Bryant R, Knaevelsrud C, Wittmann L, Rufer M, Müller J. Combining biofeedback and Narrative Exposure Therapy for persistent pain and PTSD in refugees: a pilot study. European journal of psychotraumatology. 2012; 3: 17660.

53. Palic S, Elklit A. Personality dysfunction and complex posttraumatic stress disorder among chronically traumatized Bosnian refugees. J Nerv Ment Dis. 2014; 202: 111-118.

54. Pocock NS, Suphanchaimat R, Chan CK, Faller EM, Harrigan N, Pillai V, Wickramage K. Reflections on migrant and refugee health in Malaysia and the ASEAN region: In BMC proceedings. BioMed Central. 2018; 12: 4.

55. Raghavan S, Rasmussen A, Rosenfeld B, Keller AS. Correlates of symptom reduction in treatment-seeking survivors of torture. Psychological Trauma: Theory, Research, Practice, and Policy. 2013;5(4): 377–383.

56. Rees B, Travis F, Shapiro D, Chant R. Significant reductions in posttraumatic stress symptoms in Congolese refugees within 10 days of Transcendental Meditation practice. J Trauma Stress. 2014; 27: 112-115.

57. Schultz P, Huber L, Resick P.. Practical adaptations of cognitive processing therapy with Bosnian refugees: Implications for adapting practice to a multiracial clientele. Cognitive and Behavioral Practice. 2006; 13(4): 310-321.

58. Sierre van Wyk S, Schweitzer R, Brough M, Vromans L, Murray K. A longitudinal study of mental health in refugees from Burma: The impact of therapeutic interventions. Aust N Z J Psychiatry. 2012; 46: 995-1003.

59. Stammel N, Knaevelsrud C, Schock K, Walther LC, Wenk-Ansohn M, Böttche M. Multidisciplinary treatment for traumatized refugees in a naturalistic setting: symptom courses and predictors. European journal of psychotraumatology. 2017; 8(sup2): 1377552.

60. Tay AK, Miah MAA, Khan S, Badrudduza M, Morgan K, Balasundaram S, Silove D. Theoretical background, first stage development and adaptation of a novel Integrative Adapt Therapy (IAT) for refugees. Epidemiology and psychiatric sciences. 2019; 29.

61. Tran AN, Ornelas IJ, Kim M, Perez G, Green M, Lyn MJ, Corbie-Smith G. Results from a pilot promotora program to reduce depression and stress among immigrant Latinas. Health promotion practice. 2014; 15: 365-372.

62. Weine SM, Kulenovic AD, Pavkovic I, Gibbons R. Testimony psychotherapy in Bosnian refugees: A pilot study. Am J Psychiatry. 1999; 155: 1720-1726.

63. Whitsett D, Sherman MF. Do resettlement variables predict psychiatric treatment outcomes in a sample of asylum-seeking survivors of torture? Int J Soc Psychiatry. 2017; 63: 674-685.

64. Zehetmair C, Kaufmann C, Tegeler I, Kindermann D, Junne F, Zipfel S, Nikendei C. Psychotherapeutic Group Intervention for Traumatized Male Refugees using Imaginative Stabilization Techniques–a Pilot Study in a German Reception Center. Frontiers in psychiatry. 2018; 9: 533.

65. Heeren M, Wittmann L, Ehlert U, Schnyder U, Maier T, Müller J. Psychopathology and resident status - comparing asylum seekers, refugees, illegal migrants, labor migrants, and residents. Compr Psychiatry. 2014; 55(4): 818-825.

66. Fondazione I. S. M. U. Ventitreesimo Rapporto sulle migrazioni. 2017.  Franco Angeli; 2017.

67. Norton PJ. Depression Anxiety and Stress Scales (DASS-21): psychometric analysis across four racial groups. Anxiety, Stress, and Coping. 2007; 20: 253-265.

68. Rohlof HG, Knipscheer JW, Kleber RJ. Somatization in refugees: a review. Soc Psychiatry Psychiatr Epidemiol. 2014; 49:1793-1804.

69. Pennebaker J W, Evans J F. Expressive Writing: Words that Heal: Using Expressive Writing to Overcome Traumas and Emotional Upheavals, Resolve Issues, Improve Health, and Buid Resilience. Idyll Arbor, Incorporated; 2014.
